# Application of machine learning approaches to predict seizure-onset zones in patients with drug-resistant epilepsy: a systematic review

**DOI:** 10.3389/fneur.2025.1687144

**Published:** 2025-12-01

**Authors:** Ali Haider Bangash, M. Michael Bercu, Richard W. Byrne, Spriha Pavuluri, Afshin Salehi

**Affiliations:** 1 Hhaider5 Research Group, Bronx, NY, United States; 2 Department of Neurosurgery, Montefiore Medical Center, Albert Einstein College of Medicine, Bronx, NY, United States; 3 Department of Neurosurgery, Helen DeVos Children’s Hospital, Spectrum Health, Grand Rapids, MI, United States; 4 Department of Neurosurgery, Mayo Clinic, Jacksonville, FL, United States; 5 Department of Neurology, University of Nebraska Medical Center, Omaha, NE, United States; 6 Department of Neurosurgery, University of Nebraska Medical Center, Omaha, NE, United States

**Keywords:** epilepsy, drug-resistant epilepsy, seizure-onset zone, machine learning, localization error

## Abstract

Machine learning (ML) approaches have emerged as promising tools for improving seizure-onset zone (SOZ) prediction in patients with drug-resistant epilepsy (DRE). This systematic review aimed to evaluate the application and performance of ML approaches for SOZ prediction in patients with DRE. A comprehensive search was conducted across PubMed/MEDLINE, the Cochrane Database of Systematic Reviews, and Epistemonikos databases for studies employing ML algorithms for SOZ prediction in patients with DRE. The Quality Assessment of Diagnostic Accuracy Studies version 2 (QUADAS-2) tool was adopted to assess the methodological quality and risk of bias of included studies. Data on patient demographics, data acquisition methods, ML algorithms, and performance metrics were extracted and systematically synthesized. Out of a total of 38 studies, 15 studies met the inclusion criteria, encompassing 352 patients (mean age: 28 years, 34% female population). The studies employed various ML techniques, including traditional methods such as support vector machines and advanced deep learning architectures. Performance metrics varied widely across studies, with some approaches achieving accuracy, sensitivity, and specificity values above 90%. Deep learning models generally outperformed traditional methods, particularly in handling complex, multimodal data. Notably, personalized models demonstrated superior performance in reducing localization error and spatial dispersion. However, heterogeneity in data acquisition methods, patient populations, and reporting standards complicated direct comparisons between studies. This review highlighted the potential of ML approaches, particularly deep learning and personalized models, to enhance SOZ prediction accuracy in patients with DRE. However, several challenges were identified, including the need for standardized data collection protocols, larger prospective studies, and improved model interpretability. The findings underscore the importance of considering network-level changes in epilepsy when developing ML models for SOZ prediction. Although ML approaches show promise for improving surgical planning and outcomes in DRE, their clinical utility, particularly in complex epilepsy cases, requires further investigation. Addressing these challenges will be crucial in realizing the full potential of ML in enhancing epilepsy care.

## 1 Introduction

Epilepsy affects more than 65 million people worldwide, and drug-resistant epilepsy (DRE) occurs in roughly 30% of cases (1, 2). DRE, which is defined as the failure to achieve long-term seizure freedom despite adequate trials of two appropriately chosen and tolerated antiepileptic drugregimens, represents a significant clinical challenge with profound impacts on quality of life, morbidity, and mortality (2, 3). The condition is associated with increased healthcare utilization and costs, reduced employment opportunities, and substantial psychosocial burden, and increased healthcare costs (4).

### 1.1 Clinical pathophysiology and evaluation overview of seizure-onset zones

For patients with DRE, surgical intervention often represents the most promising therapeutic option, with successful outcomes heavily dependent on accurate identification and complete resection of the seizure-onset zone (SOZ) (5). The SOZ, which is defined as the area of cortex from which clinical seizures originate, serves as the primary target for surgical intervention (6). Seizure pathophysiology in DRE varies by origin. Temporal lobe epilepsy, particularly involving mesial structures, is the most common focal epilepsy type and is often associated with hippocampal sclerosis (7). Neocortical epilepsies may arise from various regions, including frontal, parietal, and occipital lobes, frequently associated with cortical dysplasias, tumors, or post-traumatic lesions (8–10). Some patients present with multifocal origins, making surgical planning particularly challenging (11).

Clinical manifestations of seizures depend on the location of the SOZ and seizure propagation pathways. Patients may experience sensory or experiential auras (olfactory, visual, déjà vu), automatisms (lip smacking, hand fumbling), altered consciousness, and various motor manifestations ranging from subtle posturing to generalized tonic–clonic activity (12, 13). These clinical features provide important clues about seizure localization but are insufficient for precise surgical planning (14).

Current diagnostic approaches for SOZ localization employ multiple complementary modalities (15). Scalp electroencephalography (EEG) provides temporal information about seizure activity but has limited spatial resolution and reduced sensitivity to deep brain structures (16, 17). Invasive recordings through stereo electroencephalography (SEEG) (depth electrodes) or electrocorticography (ECoG) (subdural grids and strips) offer superior spatial resolution but involve surgical risks and sample only limited brain regions (18). Furthermore, structural magnetic resonance imaging (MRI) can identify epileptogenic lesions but fails to reveal any lesion in up to 30% of the cases (19). Additionally, functional neuroimaging techniques include magnetoencephalography (MEG) (measuring magnetic fields generated by neuronal activity), positron emission tomography (identifying hypometabolic regions), and functional MRI (mapping brain activity by detecting changes in blood flow) (20–22). Despite such multimodal approaches, traditional visual analysis of these diagnostic modalities is time-consuming, requires substantial expertise, and can be subject to inter-reader variability (23). Moreover, the complex spatiotemporal dynamics of epileptic networks often make precise SOZ localization challenging, potentially leading to suboptimal surgical outcomes (24).

### 1.2 Machine learning applications in seizure-onset zone prediction

Machine learning (ML) has emerged as a promising tool for enhancing the accuracy and efficiency of SOZ prediction (25). Recent advances in ML algorithms, particularly deep learning architectures, have demonstrated a remarkable ability to detect subtle patterns and relationships in complex neurophysiological data (26). These computational approaches offer the potential for automated, objective, and potentially more accurate SOZ identification than traditional methods (25).

ML methods explored in the context of epilepsy management range from traditional supervised learning approaches to advanced deep learning architectures (27). Traditional methods include support vector machine (SVM), random forest (RF), and logistic regression (LR), which typically rely on manually extracted features from neurophysiological data (28). Deep learning approaches, such as convolutional neural network (CNN) and recurrent neural network (RNN), can automatically learn hierarchical features from raw data, potentially capturing complex spatiotemporal patterns characteristic of epileptic activity (29). The application of ML across different diagnostic modalities offers unique advantages. EEG-based ML models leverage the high temporal resolution of electrophysiological recordings to detect transient epileptiform patterns (30–34). SEEG-based approaches benefit from direct recordings of deep brain structures with reduced artifact contamination (35). Moreover, MRI-based models can identify subtle structural abnormalities not apparent on visual inspection (36, 37). Multimodal approaches aim to integrate complementary information across modalities, potentially overcoming the limitations of single-modality analysis (38).

Various ML applications in epilepsy have shown encouraging results, from automated seizure detection to the prediction of surgical outcomes (39). Specifically, in SOZ prediction, ML approaches have demonstrated the ability to integrate multiple data modalities and extract relevant features that might not be apparent through conventional analysis (40). Despite these advances, several critical gaps remain in our understanding of ML applications for SOZ prediction in DRE. The relative performance of different ML approaches has not been systematically evaluated across diverse patient populations. The optimal combination of input features and algorithmic architectures also remains unclear. Moreover, the clinical validation and implementation of these methods in real-world settings require further investigation. A comprehensive systematic review of ML approaches for SOZ prediction is, therefore, timely and necessary to guide both technical development and clinical application. Such analysis could inform the development of more accurate and reliable SOZ prediction methods, potentially improving surgical outcomes and ultimately the lives of patients with DRE.

## 2 Methods

### 2.1 Search strategy

The systematic review was carried out in accordance with the Preferred Reporting Items for Systematic Review and Meta-Analysis (PRISMA) guidelines (41), with a prospectively developed study protocol (PSP) guiding the objectives, search strategy, and planned analyses. As this was a systematic review of primary studies, ethical approval was not required. We aimed to explore the application of ML for predicting SOZ in patients with DRE. Moreover, we also aimed to compare the performance metric values of the ML algorithms used. The literature search, study selection, methodological quality assessment, and synthesis of results were undertaken by AHB and authenticated by AS.

The literature search was carried out through PubMed/MEDLINE, Cochrane Database of Systematic Reviews (CDSR), and Epistemonikos in accordance with the PSP. The reference lists of the included studies were also searched for relevant publications. The search strategy included the terms “seizure-onset zone,” “electroencephalography” [MeSH Terms], and “machine learning” [MeSH Terms]. A detailed version of the strategy is provided in the Supplementary material.

### 2.2 Study selection

The cohort of eligible articles was reviewed by evaluating the abstracts and full texts, as required. Studies were included if they met the predetermined inclusion criteria: (1) studies that adopted ML algorithms for predicting the SOZ in patients with DRE; (2) original research articles published in peer-reviewed journals; and (3) published from database inception until August 19, 2024. Studies were excluded if they: (1) did not use ML algorithms for SOZ prediction; (2) focused on patient populations other than patients with DRE or included mixed patient populations where epilepsy data could not be separately extracted; (3) lacked methodological descriptions or contained insufficient data to assess the ML approach; or (4) were review articles, editorials, conference abstracts, or letters to the editor. The updated PRISMA flow diagram was adopted to represent the study selection process transparently.

Manual extraction of the required data, in accordance with the predetermined “Characteristics of studies” table, was carried out. The extracted variables included the author and year, study design, and datasets used. The number of patients, along with age and percentage of the female population, were also extracted. Moreover, the methods of data acquisition, algorithmic models used, and the reported performance metrics values were also collected.

### 2.3 Quality assessment and risk of bias

Using the Cochrane Review Manager (version 5.4.1), the Quality Assessment of Diagnostic Accuracy Studies version 2 (QUADAS-2) tool was adopted to assess the methodological quality and risk of bias of the included studies (42). The QUADAS-2 analysis enabled the assessment of the overall risk of bias, which was stratified into four domains: (1) description of methods of patient selection (patient selection); (2) conduction and interpretation of the index test (index test); (3) description of the reference standard (reference standard); (4) description of the patients excluded from test induction and of the interval, as well as any interventions, between index tests and the reference standard (flow and timing) (42).

### 2.4 Statistical analysis

An exploratory data analysis was performed. The categorical variables were expressed as percentages of the total. The MedCalc statistical software (v. 20.215) was used to perform the analyses.

## 3 Results

**Figure 1:**
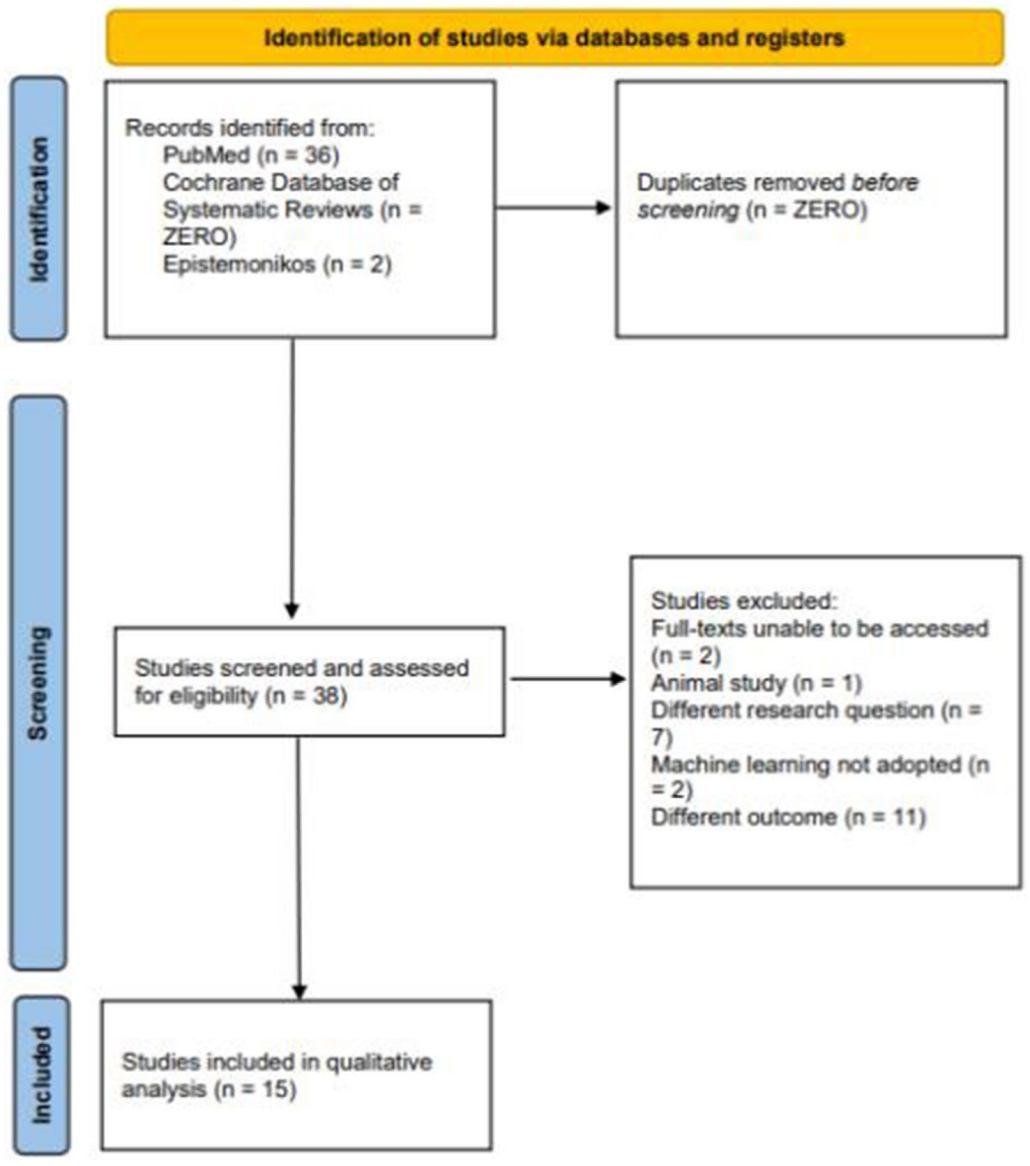
PRISMA flow chart illustrating the search, selection, and inclusion of studies exploring machine learning for predicting seizure-onset zone(s) in patients with drug-resistant epilepsy.

A systematic search of PubMed/MEDLINE, CDSR, and Epistemonikos identified 38 articles. After screening abstracts and full texts, 15 studies met the inclusion criteria and were included in the review (Table 1) (43–57). The details of the excluded studies are provided in the Supplementary material, and the reasons for exclusion are summarized in the PRISMA flow diagram (Figure 1).

**Table 1:** Characteristics of studies exploring machine learning for predicting seizure-onset zone(s) in patients with drug-resistant epilepsy.

Study and year	Country	Type of study	Datasets	Age	Number of patients	% Female population	Extent of intervention (lesionectomy, larger resection, disconnection, laser ablation)	Postoperative outcomes	Neurostimulation (RNS/DBS)	Neurostimulation outcomes	Length of follow-up	Method of data acquisition
Balaji SS et al., 2024(43)	USA	Retrospective	HUP iEEG epilepsy dataset	Not specified	58	Not specified	Lesionectomy or laser ablation (specifics not provided)	Subgroup analysis of Engel I	Not adopted	–	Not specified	Intracranial electroencephalogram (iEEG) recordings were obtained using subdural grids, strips, and depth electrodes— electrocorticography (ECoG) or stereoelectroencephalography (SEEG). The number of electrodes and the sampling frequency varied by subject and electrode type. The iEEG data during seizure onset were used to estimate effective connectivity measures. For constructing classifiers, features were extracted from preictal, ictal, and interictal time windows. The study focused on a 30-s window centered around seizure onset for feature extraction. The study focused on patients who achieved seizure-free status post-surgery (Engel class I), resulting in a subset of 28 individuals. The data were preprocessed to remove artifacts and unreliable electrodes, with the recordings resampled and filtered for consistency.
Sun R et al., 2023 (44)	USA	Retrospective	University of Pittsburgh Medical Center and Minnesota Epilepsy Group (shared as supplementary materials)	37.46 ± 15.47 years	29	51.72%	Lobectomy adopted in 19 patients Amygdalohippocampectomy adopted in two patients Sublobar resection adopted in three patients Lesionectomy of epileptogenic focus undertaken in three patients AVM removal undertaken in one patient Cortical dysplasia resection adopted in one patient	Engel I for all patients	Not adopted	–	At least 12 months	Magnetoencephalography (MEG) and iEEG monitoring MEG sessions lasted between 20 and 60 min, with recordings sampled at 1000–1017 Hz. Two MEG systems were used: Magnes 2,500 WH with 148 magnetometers (Minnesota Epilepsy Group). Elekta Neuromag Vector View with 102 magnetometers and 204 planar gradiometers (UPMC).
Johnson GW et al., 2022 (45)	USA	Retrospective	Vanderbilt University Medical Center (shared as an in-manuscript table)	34 ± 12.06 years	10	70%	Selective amygdalohippocampectomy adopted in three patients Anterior temporal lobectomy adopted in three patients	Amygdalohippocampectomy subgroup: Engel class IA for 66.7% (2 of 3) patients Engel class ID for 33.3% (1 of 3) patients Lobectomy subgroup: Engel class IA in 100% (*n* = 3) patients	Bilateral responsive neurostimulation adopted in four patients	>50% reduction: 50% (2 of 4) patients <50% reduction: 50% (2 of 4) patients	Mean: 15.4 months	Single-pulse electrical stimulation (SPES) was performed on every adjacent bipolar pair of SEEG contacts implanted in the gray matter of these patients. The stimulation involved a 10-s, 1-Hz, 300-ms biphasic pulse at 3.0 mA, with recordings sampled at 512 Hz. The SEEG data were filtered using MATLAB’s filtfilt function with specific Butterworth filter passbands (1–59 Hz, 61–119 Hz, 121–150 Hz) and then parsed into 900-ms epochs after each stimulation, resulting in over 500,000 preprocessed epochs for model training.
Zhao X et al., 2022 (46)	Japan	Retrospective	Epilepsy Center of Juntendo University (shared as in-manuscript table)	22.67 ± 11.08 years	6	40%	Cortical dysplasia resection adopted in all six patients	Engel class IA for 83.3% (5 of 6) patients Engel class IIIA for 16.7% (1 of 6) patients	Not adopted	–	Mean: 51 months	iEEG data were collected from six patients using subdural electrodes placed over the cortical surface. The recordings were sampled at 2000 Hz and segmented into 10-s epochs for analysis. The data were split into seizure-onset zone (SOZ) and non-SOZ channels as identified by clinical experts. A Butterworth bandpass filter (0.5–900 Hz) was applied to the synthetic iEEG samples generated during data augmentation.
Jeong JW et al., 2022 (47)	USA	Retrospective	Wayne State University (not publicly shared)	9.9 ± 5.6 years	41	46.34%	Two-stage epilepsy surgery (specifics not provided)	ILAE class 1 for all patients	Not adopted	–	At least 12 months	iEEG using subdural grids and multimodal MRI data were collected from pediatric patients with drug-resistant epilepsy (DRE). The MRI data included T1-weighted, T2-weighted, FLAIR, and DWI/DWIC sequences acquired on a GE Signa 3 T scanner with an 8-channel head coil. Imaging parameters included a T1-weighted TR of 6.1 ms and TE of 2.4 ms, with slice thicknesses of 1.2–5 mm depending on the modality. Cortical parcellation and surface-based laminar analyses were applied to extract gray and white matter surface markers, including relative intensity values. Diffusion tractography was performed to analyze intra-hemispheric white matter connectivity.
Bhanot N et al., 2022 (48)	India	Retrospective	NIMHANS (shared as an in-manuscript table)	24.2 ± 10.59 years	15	40%	Specifics not provided	Not reported	Not adopted	–	Not specified	MEG recordings were collected using a 306-channel Elekta Neuromag® TRIUX system with sampling frequencies of 2 or 5 kHz, bandpass filtered (0.1–100 Hz), and down-sampled to 250 Hz. EEG data were also collected alongside MEG for comprehensive analysis. Preprocessing involved artifact removal and head movement correction using Elekta MaxFilter software. Data were segmented into 1-s, non-overlapping epochs, yielding more than 11,000 labeled MEG and EEG epochs. Features extracted included short-time permutation entropy (STPE), gradient of STPE, short-time energy, and short-time mean, which were used for model training.
Craley J et al., 2022 (49)	USA	Retrospective	Johns Hopkins Hospital and University of Wisconsin-Madison (shared as supplementary materials)	JHH dataset: 35.7 ± 16.8 years; UWM dataset: Mean: 13 ± 3.1 years	JHH dataset: 34 patients; UWM dataset: 15 patients	JHH dataset: 52.94%; UWM dataset: 33.3%	Focal resection (specifics not provided)	Not reported	Not adopted	–	Not specified	EEG recordings were obtained using a Nihon Kohden system at a sampling rate of 200 Hz, filtered with a 60-Hz notch filter, a 70-Hz low-pass filter, and a 0.016-Hz high-pass filter. Data were band-passed between 0.5 and 30 Hz and normalized. EEG signals were parsed into 1-s non-overlapping windows for input to the model, resulting in 201 seizure recordings from 34 patients. Data preprocessing involved artifact removal by thresholding recordings at two standard deviations from the mean.
Charupanit K et al., 2020 (50)	USA	Retrospective	University of California, Irvine (shared as an in-manuscript table)	38.2 ± 16.9 years	11	45.45%	Lobectomy adopted in eight patients	Engel class IA for 12.5% (1 of 8) patients managed with lobectomy Engel class IB for 25% (2 of 8) patients managed with lobectomy Engel class ID for 12.5% (1 of 8) patients managed with lobectomy Engel class IIB for 12.5% (1 of 8) patients managed with lobectomy Engel class IIIA for 37.5% (3 of 8) patients managed with lobectomy.	Responsive neurostimulation adopted in three patients	Engel class IIA for 33% (1 of 3) patients managed with RNS Engel class IIIA for 33% (1 of 3) patients managed with RNS Engel class IVB for 33% (1 of 3) patients managed with RNS	Not specified	iEEG recordings were collected from 11 patients with medically refractory epilepsy at the University of California, Irvine Medical Center. Data were sampled at 2 kHz (10 subjects) or 5 kHz (1 subject). SEEG data were preprocessed using bandpass filters and segmented into 3-min interictal data epochs. An anomaly detection algorithm and root-mean-square detectors were used to identify high-frequency events, yielding more than 598 SEEG segments for model training and classification between SOZ and non-SOZ channels.
Prasanna et al., 2020 (51)	India, Iraq, Saudi Arabia, Spain	Retrospective	University of Bonn (UOB) and Bern–Barcelona (BB) public datasets	Not specified	BB Dataset: 5 patients; UOB dataset: Not specified	Not specified	Specifics not provided	BB Dataset: 60% (3 of 5) patients were completely cured 40% (2 of 5) patients experienced seizure sensations postoperatively UOB dataset: Not specified	Not adopted	–	Not specified	The study used two publicly available EEG datasets for the automatic classification of non-focal class (NFC) and focal class (FC) EEG signals. The first dataset, the UOB EEG dataset, contains 500 single-channel EEG files categorized into five classes (A, B, C, D, and E). Only classes C and D, representing interictal EEG signals from focal epileptic subjects, were included in the analysis. Each EEG signal had a duration of 23.6 s and was sampled at a frequency of 173.61 Hz, with artifacts removed by visual inspection. The second dataset, the BB dataset, comprises iEEG recordings from five patients with temporal lobe epilepsy. These recordings were divided into NFC and FC groups. Each recording had 10,240 samples with a sampling rate of 512 Hz, and the signals were preprocessed using a bandpass filter to remove artifacts.
Wang Z et al., 2020 (52)	China	Retrospective	Shenyang University of Technology (not shared)	Not specified	6	Not specified	Specifics not provided	All patients were seizure-free postoperatively.	Not adopted	–	Not specified	SPES was conducted on each adjacent bipolar pair of SEEG contacts implanted in the gray matter of patients with DRE. The stimulation involved a 10-s, 1-Hz, 300-ms biphasic pulse at 3.0 mA, with recordings sampled at 512 Hz. The SEEG data were filtered using MATLAB’s filtfilt function with specific Butterworth filter passbands (1–59 Hz, 61–119 Hz, and 121–150 Hz) and segmented into 900-ms epochs following each stimulation, yielding more than 500,000 preprocessed epochs for model training.
Sumsky SL et al., 2019 (53)	USA	Retrospective	University of Connecticut (shared as an in-manuscript table)	24.5 ± 9.95 years	14	35.71%	Resection surgery (specifics not provided)	Engel/ILAE class I for 57% (8 of 14) patients Engel/ILAE class IV for 14% (2 of 14) patients Engel/ILAE class V for 7% (1 of 14) patients*	Not adopted	–	12 months	Fourteen de-identified patients with DRE from the National Institutes of Neurological Disorders and Stroke iEEG Portal were included. Patients underwent continuous iEEG recordings for 2–7 days, sampled at 512 Hz. Data were high-pass filtered (8th order Butterworth, 80 Hz cut-off) and referenced to a common average. iEEG time series were segmented into 10-min epochs, and ripple events were automatically detected using a validated algorithm.
Varatharajah Y et al., 2018 (54)	USA	Retrospective	Mayo Clinic (shared as supplementary materials)	46.5 ± 18.8 years	82	41.46%	Specifics not provided	ILAE class I for 29% (24 of 82) patients ILAE class II for 13% (11 of 82) patients ILAE class III for 5% (4 of 82) patients ILAE class IV for 10% (8 of 82) patients ILAE class V for 12% (10 of 82) patients ILAE class VI for 2% (2 of 82) patients^¶^	Not adopted	–	Not specified	Continuous 2-h interictal iEEG segments were selected from 82 patients, with 4,966 electrodes implanted, including 911 within SOZs. Data were reviewed using MATLAB, and artifact-containing segments were excluded. The recordings were filtered to remove 60-Hz power-line artifacts. Data were segmented into 3-s epochs, and biomarkers (high-frequency oscillations, interictal epileptiform discharges, and phase-amplitude coupling) were detected and clustered to classify channels as normal or abnormal. Binary observations of biomarkers were summarized over 10-min windows, yielding 36 local biomarker rates per channel, which were used for support vector machine (SVM) classification.
Elahian B et al., 2017 (55)	USA	Retrospective	Le Bonheur Children’s Hospital (shared as an in-manuscript table)	23 ± 9 years	10	30%	Specifics not provided	Engel class I for 60% (6 of 10) patients Engel class III for 10% (1 of 10) patients Engel class IV for 30% (3 of 10) patients	Not adopted	–	Mean: 13.9 months	Subdural ECoG recordings were collected using a standard clinical video EEG system at 1 kHz after bandpass filtering (0.1–300 Hz). Bipolar montages were used, excluding artifact channels. Phase-locking value (PLV) was computed to measure cross-frequency coupling between low-frequency phases (4–30 Hz) and high gamma frequency amplitudes (80–150 Hz) using the Hilbert transform. PLV was calculated during a 5-min interictal window and pre- and post-ictal intervals. Extracted features included PLV mean, peak, duration, and power, which were used in a logistic regression classifier to identify SOZ electrodes.
Dian JA et al., 2015 (56)	Canada	Retrospective	University of Toronto (not shared)	16–56 years	6	33.30%	Resection (specifics not provided)	Engel class I for 17% (1 of 6) patients Engel class III for 17% (1 of 6) patients Engel class IV for 17% (1 of 6) patients^§^	Not adopted	–	Not specified	iEEG recordings were collected from six patients undergoing epilepsy resection surgery using PMT cortical electrodes (10.0 mm interelectrode spacing, 3.0 mm diameter). Electrodes were placed on the frontal and temporal lobes and sampled at 2 kHz after amplification. Seizure onset and offset times were identified from clinical notes and video recordings. Preprocessing included electrode grid transformation and filtering using a 50-Hz FIR notch filter. Empirical mode decomposition and its enhanced variant were used to decompose the signals into intrinsic mode functions (IMFs) within low (1–80 Hz) and high-frequency (80–400 Hz) ranges. Extracted features from these IMFs included power, RMS power, mean, variance, skewness, kurtosis, line length, zero crossings, non-linear energy, activity, mobility, and complexity, generating a unique feature vector for each channel and time window.
Zhu G et al., 2015 (57)	Australia	Retrospective	UOB and BB public datasets	Not specified	BB Dataset: 10 patients; UOB dataset: Not specified	Not specified	Specifics not provided	Not reported	Not adopted	–	Not specified	Two EEG databases were analyzed. The first database, described by Andrzejak et al., includes five datasets (A-E) sampled at 173.61 Hz with a 0.53–40-Hz bandpass filter. It contains single-channel EEG signals from healthy volunteers (Sets A and B) and epileptic patients (Sets C, D, E), with each set comprising 100 signals of 4,097 data points. The second database, the BB EEG database, includes two sets: one from the epileptogenic zone (Set F) and another from non-epileptogenic areas (Set N). The sample rate was 512 Hz for fewer than 64 channels and 1,024 Hz otherwise. Each recording contained signals from a focal EEG channel and a neighboring channel, with 10,240 data points per signal. This database was subdivided into two sets: #50 (100 recordings) and #750 (4,500 recordings). Features were extracted based on delay permutation entropy and applied to an MSK-means classifier, with results compared to those from standard K-means and SVM classifiers.

*Twenty-one percent (3 of 14) of patients were not surgically treated following presurgical evaluation. ^¶^Postoperative outcomes were not reported for 28% (23 of 82) of patients. ^§^Postoperative outcomes were not reported for 50% (3 of 6) of patients. iEEG, intracranial electroencephalogram; EcoG, electrocorticography; SEEG, stereoelectroencephalography; MEG, magnetoencephalography; SPES, single-pulse electrical stimulation; SOZ, seizure-onset zone; DRE, drug-resistant epilepsy; STPE, short-time permutation entropy; NFC, non-focal class; FC, focal class; UOB, University of Bonn; BB, Bern–Barcelona; SVM, support vector machine; PLV, phase-locking value; IMF, intrinsic mode function.

### 3.1 General patient demographics and characteristics of studies

All included studies were retrospective, published between 2015 and 2024, and included a total of 352 patients (mean age 28 ± 10.7 years; 34% female population) (44–50, 53–56). Most studies were conducted in the United States (60%, *n* = 9) (43–45, 47, 49, 50, 53–55). In addition, 73% (*n* = 11) of the studies did not report the specific surgical intervention type at the individual patient level (43, 47–49, 51–57), whereas 27% (*n* = 4) of the studies included only Engel/ILAE class I patients (43, 44, 47, 52) and 20% (*n* = 3) of the studies did not report postoperative outcomes (48, 49, 57). Neurostimulation was reported in only 13% (*n* = 2) of the studies (45, 50).

### 3.2 Quality assessment and risk of bias

**Figure 2:**
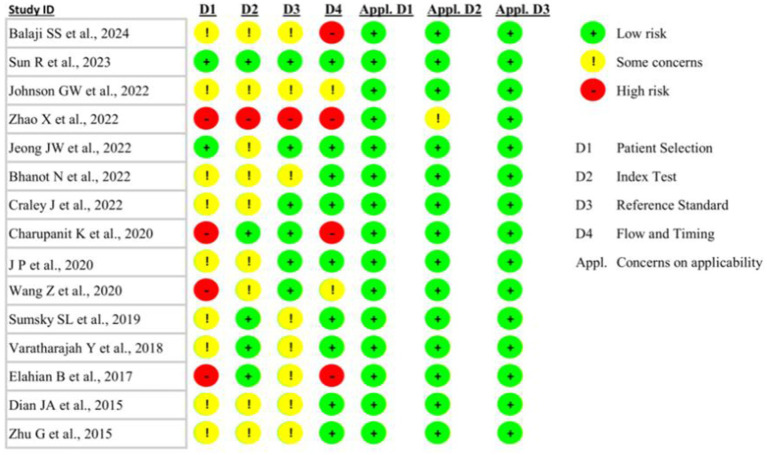
Quality Assessment of Diagnostic Accuracy Studies—version 2 (QUADAS-2) analysis of the included studies exploring machine learning for predicting seizure-onset zone(s) in patients with drug-resistant epilepsy.

Upon implementing the QUADAS-2 tool to assess the risk of bias in the methodology and applicability of the included studies, 13% (*n* = 2) of the studies were found to have a low risk of bias in the patient selection domain (44, 47) (Figure 2). The study by Zhao et al. had a high risk of bias across all four domains (46), whereas 27% (*n* = 4) of the studies were found to have a high risk of bias in the “Flow and Timing” domain, specifically regarding whether all patients received the same reference standard and whether there was an appropriate interval between the index test and reference standard (43, 46, 50, 55) (Figure 2). With the exception of a single study, which exhibited an unclear concern for the applicability of the diagnostic test under evaluation (the “index test”) (46), all other studies were found to have a low risk of concern across all three applicability domains (Figure 2).

### 3.3 Datasets and methods of data acquisition

Only 20% (*n* = 3) of the studies adopted publicly available datasets (43, 51, 57) (Table 1). Overall, 80% (*n* = 12) of the studies used private datasets, and 40% (*n* = 6) of the studies shared their dataset as an in-manuscript table (45, 46, 48, 50, 53, 55). EEG was found to be the most common source of data acquisition, adopted by 87% (*n* = 13) of the studies (43, 44, 46–51, 53–57). Moreover, SEEG was adopted by 20% (*n* = 3) of the studies (43, 45, 52), whereas ECoG (43, 55) and MEG (44, 48) were adopted by 13% (*n* = 2) of the studies each (Table 1).

### 3.4 Algorithmic models

The included studies investigated a wide range of ML approaches, from conventional supervised learning methods to sophisticated deep learning (DL) architectures for predicting the SOZ in patients with DRE (Table 2).

**Table 2:** Algorithmic architecture and prediction performance reported by studies exploring machine learning for predicting seizure-onset zone(s) in patients with drug-resistant epilepsy.

Study and year	Algorithmic models	Prediction performance (results)
Balaji SS et al., 2024 (43)	Graph feature-based supervised machine learning (ML) models: These models utilized graph centrality measures extracted from effective connectivity (EC) graphs as features for classification. The specific supervised ML algorithms applied include the following: Support vector machine (SVM) with radial basis function (RBF) kernels. Multilayer perceptron (MLP). EC measures: the study employed three model-free EC measures to generate directed graphs: Directed information (DI). Mutual Information-Guided Granger Causality Index (MI-GCI). Frequency-domain convergent cross-mapping (FD-CCM). These measures are used to capture the causal relationships and dynamic network interactions in the brain, which are crucial for identifying the seizure-onset zone (SOZ) in epilepsy patients	DI-based features: Accuracy: 92.12% Sensitivity (SS): 85.3% Specificity (SP): 92.8% Area under curve (AUC): 0.89 Best classifier: MLP with 10% sparsity MI-GCI-based features: Accuracy: 95.72% Sensitivity (SS): 89.74% Specificity (SP): 96.32% AUC: 0.90 Best classifier: SVM with RBF kernel at 90% sparsity FD-CCM-based features: Accuracy: 94.10% Sensitivity (SS): 92.3% Specificity (SP): 94.25% AUC: 0.93 Best classifier: SVM with RBF kernel at 10% sparsity Each EC measure demonstrated strong classification performance, with FD-CCM-based features showing the highest AUC value, indicating superior discrimination between SOZ and non-SOZ electrodes.
Sun R et al., 2023 (44)	The study adopted a deep learning (DL)-based source imaging framework (DeepSIF) for electromagnetic source imaging. It utilizes deep neural networks trained on data generated by neural mass models. Two specific models were developed: Generic DeepSIF (GDeepSIF): used a template head model for training. Personalized DeepSIF (PDeepSIF): incorporated personalized head geometry derived from individual patients’ MRIs to improve performance.	GDeepSIF: Sublobar concordance rate (CR): 83% Sublobar sensitivity: 66% Sublobar specificity: 97% Localization error (LE): 24.86 ± 10.40 mm Spatial dispersion (SD): 21.90 ± 19.03 mm F1 score (harmonic mean of precision and recall): not explicitly mentioned, but can be inferred from sensitivity and specificity. PDeepSIF: Sublobar CR: 93% Sublobar sensitivity: 77% Sublobar specificity: 99% LE: 15.78 ± 5.53 mm SD: 8.19 ± 8.14 mm F1 score: significantly higher than GDeepSIF due to better balance between sensitivity and specificity. Comparison to conventional methods: PDeepSIF demonstrated superior performance compared to conventional methods like LCMV, sLORETA, and CMEM, especially in: SD: 8.19 ± 8.14 mm for PDeepSIF, compared to 33.27 ± 16.19 mm (LCMV), 29.10 ± 14.66 mm (sLORETA), and 29.15 ± 18.66 mm (CMEM). SOZ LE: 15.78 ± 5.53 mm for PDeepSIF, compared to 25.94 ± 7.07 mm (LCMV), 22.89 ± 6.14 mm (sLORETA), and 31.35 ± 15.86 mm (CMEM).
Johnson GW et al., 2022 (45)	Multichannel one-dimensional convolutional neural network (CNN) for the classification of SOZ based on stereoelectroencephalography (SEEG) cortico-cortical evoked potentials. The specific CNN model was a modified version of the multiscale-1D-ResNet. The CNN was trained on 500,000 unique post-stimulation SEEG epochs to determine whether an SOZ had been stimulated. The model utilized a weighted binary cross-entropy loss function and employed a leave-one-patient-out testing strategy to validate its performance.	Sensitivity: 78.1% (95% CI 67.8–88.4%) Specificity: 74.6% (95% CI 68.7–80.5%) Youden Index (YI): 52.7 (95% CI 43.7–61.8) Additional metrics: Performance on different time windows: 0–350 ms window: Sensitivity: 74.0% (95% CI 63.3–84.7%) Specificity: 78.5% (95% CI 75.9–81.1%) YI: 52.5 (95% CI 42.1–62.9) 50 ms sliding window: Sensitivity peaked in the 100–150 ms window. Specificity peaked in the 0–50 ms window. Classification performance: Unilateral mesial temporal lobe epilepsy (TLE): Sensitivity for ipsilateral SOZ: 91.5% (95% CI 89.7–93.3%) False-positive rate (FPR) for contralateral (non-SOZ): 35.1% (95% CI 16.7–53.5%) Bilateral mesial TLE: Sensitivity for left mesial temporal SOZ: 68.9% (95% CI 58.7–79.1%) Sensitivity for right mesial temporal SOZ: 67.9% (95% CI 45.4–90.4%) Neocortical temporal SOZs: Sensitivity: 64.4% (95% CI 44.3–84.5%) FPR for non-SOZs: 26.0% (95% CI 19.7–32.3%) These metrics indicate that the DL model showed robust performance in classifying SOZs based on SEEG data with a significant degree of accuracy across different epilepsy types.
Zhao X et al., 2022 (46)	A one-dimensional CNN used to classify SOZ and non-SOZ data, combined with various augmentation methods and loss functions: Data augmentation methods: EEGAug: generates synthetic samples by transforming minority class intracranial electroencephalogram (iEEG) data into the frequency domain, recombining different frequency bands, and converting them back to the time domain to achieve a balanced dataset. Synthetic minority over-sampling technique (SMOTE): generates synthetic samples by interpolating between minority class samples along the line segments joining the sample and its k-nearest neighbors (KNN). Adaptive synthetic sampling (ADASYN): a dynamic sampling method that generates synthetic samples for minority class data based on their difficulty level, focusing on samples near the decision boundary. Loss functions: Class-balanced focal loss: adjusts the weights of minority and majority class data by dynamically scaling the cross-entropy loss, focusing learning on harder examples. Cross-entropy: standard loss function used for binary classification tasks.	Data augmentation methods (EEGAug, SMOTE, ADASYN): Accuracy: varies across patients, ranging approximately 0.85–0.99 Precision: varies across patients, ranging approximately 0.30–0.95 Recall: varies across patients, ranging approximately 0.30–0.90 Specificity: consistently high across patients, ranging approximately 0.90–0.99 F1 score: varies across patients, ranging approximately 0.40–0.90 Matthews correlation coefficient (MCC): varies across patients, ranging approximately 0.40–0.90 Cohen’s kappa (CK): varies across patients, ranging approximately 0.40–0.90 Class-balanced focal loss: Performance metrics similar to data augmentation methods, with slight variations
Jeong JW et al., 2022 (47)	msResNet (multiscale residual neural network): a DL model designed to non-invasively localize the SOZ in children with drug-resistant epilepsy using multimodal MRI data. The model employs multiple branches of convolution filters (1 × 3 and 1 × 7) to capture both coarse- and fine-scale features. Data augmentation: the study used artificial augmentation techniques, including interpolation across nearest neighbors with Gaussian noise addition, to balance class instances. Loss function: utilizes cross-entropy loss minimized via the Adam optimizer to differentiate SOZ from non-SOZ nodes. Comparative models: the performance of msResNet is compared with other ML models such as KNN, random forest, logistic regression (LR), MLP, conventional CNN, and ResNet18.	Model cohort (5-fold cross-validation [CV]): Training set accuracy: 0.99 Test set accuracy: 0.96 Lesional MRI patients: Sensitivity: 0.92 Specificity: 0.98 Accuracy: 0.97 Non-lesional MRI patients: Sensitivity: 0.91 Specificity: 0.96 Accuracy: 0.94 Validation cohort (msResNet performance): Lesional MRI patients: Sensitivity: 0.64 Specificity: 0.85 Balanced accuracy: 0.75 Non-lesional MRI patients: Sensitivity: 0.56 Specificity: 0.78 Balanced accuracy: 0.67 Comparison to other methods: msResNet outperformed MLP, CNN, and ResNet18 Improvement in balanced accuracy: Lesional MRI: Up to 47% (*vs*. MLP), 37% (*vs*. CNN), 44% (*vs*. ResNet18) Non-lesional MRI: Up to 16% (*vs*. MLP), 8% (*vs*. CNN), 12% (*vs*. ResNet18) Seizure onset likelihood (μi) performance: Effect size (Cohen’s d): Model cohort: 1.92 Validation cohort: 1.21 SOZ localization (μi > 0.5): True positive rate: 0.92 False negative rate: 0.08 Combined performance with iEEG markers (AUC): Non-lesional MRI group: iEEG alone: 0.56–0.62 iEEG + μi: 0.64–0.65 (5–15% improvement) Lesional MRI group: iEEG alone: 0.70–0.77 iEEG + μi: 0.70–0.77 (no improvement) This study demonstrates strong performance in the model cohort and moderate reproducibility in the validation cohort, with better results for lesional MRI patients compared to non-lesional MRI patients. The msResNet outperformed other ML methods, and the combination of the proposed MRI marker with iEEG markers showed improvement in non-lesional cases.
Bhanot N et al., 2022 (48)	RUSBoost algorithm: an ML algorithm used for seizure detection and epileptogenic zone localization from heavily skewed magnetoencephalography (MEG) data. It combines random under-sampling with AdaBoost to handle class imbalance effectively. Feature extraction: utilizes four statistical features derived from MEG data—short-time permutation entropy (STPE), gradient of STPE (GSTPE), short-time energy (STE), and short-time mean (STM)—to distinguish between ictal and interictal periods. CV: a 5-fold CV approach was used to train and evaluate the model’s performance. Localization approach (LA): region-specific classifications were performed by segmenting the data into eight brain regions and applying the RUSBoost classifier separately to localize the epileptogenic zone.	Whole-head seizure detection: Accuracy: 93.4% Sensitivity: 93% Specificity: 93% AUC: 0.97 Region-specific classifications for epileptogenic lobe localization: All regions showed high accuracies, sensitivities, and specificities (exact values not provided) Minimum AUC across all regions: 0.95 Feature vector performance: (a) STPE: Showed a clear difference between ictal and interictal periods (b) GSTPE: Slight change in distribution during the ictal period ML model able to distinguish ictal and interictal data (c) STE: Significant increase during the ictal period (d) STM: Slight increase during ictal period AUC analysis: AUC for ictal data detection: ~97% The study demonstrates high performance in both whole-head seizure detection and region-specific epileptogenic lobe localization. The various feature vectors (STPE, GSTPE, STE, and STM) showed distinct patterns during ictal periods, contributing to the effective classification. The high AUC values (0.97 for whole-head and a minimum of 0.95 for region-specific) indicate strong discriminative power of the algorithm, even with heavily skewed data.
Craley J et al., 2022 (49)	SZTrack algorithm: a DL model combining CNN and bidirectional long short-term memory (BLSTM) for seizure detection and onset zone prediction using EEG data. It tracks seizure activity on a per-electrode basis to capture spatiotemporal propagation. Feature extraction: utilizes CNN encodings to generate hidden representations from 1-s windows of EEG signals, followed by BLSTM for tracking temporal dependencies. CV: leave-one-patient-out CV (LOPO-CV) was used for model evaluation. LA: localizes seizure onset at the hemisphere and lobe level by aggregating individual electrode predictions based on coarse annotations of SOZ.	Johns Hopkins Hospital Dataset (Main dataset): (a) Seizure detection: SZTrack: AUC: 0.895 Sensitivity: 0.835 (seizure level) CNN-BLSTM (best-performing baseline): AUC: 0.899 Sensitivity: 0.919 (seizure level) (b) Lateralization (hemisphere identification): SZTrack: Highest average accuracy: 0.826 (at λsz = 0.6) Outperformed No-BLSTM and TGCN baselines (c) Lobe classification: SZTrack: Highest average accuracy: 0.587 (at λsz = 0.6) TGCN (best performing): Highest average accuracy: 0.605 (at λsz = 0.1) (d) Combined lateralization and lobe classification: Correct in both hemisphere and lobe: 20/34 patients (58.8%) Correct in either hemisphere or lobe: 14/14 remaining patients (100% of remaining) University of Wisconsin-Madison (UWM) Dataset (Generalization dataset): (a) Seizure detection: SZTrack: AC: 0.813 Sensitivity: 0.639 (seizure level) CNN-BLSTM: AUC: 0.857 Sensitivity: 0.523 (seizure level) (b) Localization (preliminary study on one LOPO-CV fold): Correct in both hemisphere and lobe: 8/15 patients (53.3%) Correct in either hemisphere or lobe: 5/15 patients (33.3%) Incorrect in both: 2/15 patients (13.3%) The study demonstrates strong performance in seizure prediction, particularly with the SZTrack model. It shows good generalization capabilities when applied to a different dataset (UWM) without fine-tuning. The model’s ability to visualize seizure spread patterns aligns well with clinical annotations, suggesting potential clinical utility.
Charupanit K et al., 2020 (50)	Amplitude-based high-frequency oscillation (HFO) Detection: utilizes two automated algorithms (ML-based anomaly detection and root-mean-square (RMS)-based detection) to identify high-frequency oscillations (HFOs) from iEEG recordings for SOZ classification. Feature extraction: focuses on amplitude and rate of HFOs, with amplitude offering more consistent classification of SOZ *versus* non-SOZ channels. CV: performance assessed through AUC and sensitivity analysis for each subject, with leave-one-subject-out testing. LA: differentiates SOZ from non-SOZ by analyzing amplitude stability over time, with HFO amplitude showing superior classification accuracy and consistency.	Event detection: SOZ channels: 6,208 anomalous high-frequency activity (aHFA) and 14,008 conventional HFO (cHFO) (598 segments) Non-SOZ channels: 15,193 aHFA and 7,179 cHFOs (1,336 segments) Event rates: cHFO rate in SOZ: 8.2 ± 4.2 per minute cHFO rate in non-SOZ: 1.9 ± 2.7 per minute Event amplitudes: aHFA amplitude in SOZ: 39.7 ± 28.8 μV aHFA amplitude in non-SOZ: 7.2 ± 8.7 μV cHFO amplitude in SOZ: 37.0 ± 29.4 μV cHFO amplitude in non-SOZ: 6.4 ± 8.1 μV Individual subject classification (AUC analysis): aHFA amplitude: Mean AUC: 0.960 Mean sensitivity: 93.7% Mean FPR: 6.1% cHFO amplitude: Mean AUC: 0.948 Mean sensitivity: 94.1% Mean FPR: 6.2% cHFO rate: Mean AUC: 0.905 Mean sensitivity: 84.9% Mean FPR: 13.7% Group-level classification: (a) Segment-based AUC: aHFA amplitude AUC: 0.946 cHFO amplitude AUC: 0.942 cHFO rate AUC: 0.880 (b) Channel-based AUC: aHFA amplitude AUC: 0.956 cHFO amplitude AUC: 0.952 cHFO rate AUC: 0.911 Robustness to detection threshold changes: Amplitude metrics (aHFA and cHFO) showed more stable AUC values across different thresholds compared to rate metrics. In summary, this study demonstrates that amplitude-based metrics (both aHFA and cHFO) outperform rate-based metrics in classifying SOZ and non-SOZ channels. Amplitude measurements showed higher AUC values, better sensitivity, lower FPRs, and greater robustness to changes in detection thresholds compared to rate measurements. These findings suggest that amplitude-based metrics may be more reliable for SOZ prediction.
Prasanna et al., 2020 (51)	Algorithmic models: Fast Walsh–Hadamard transform (FWHT) and artificial neural network (ANN): the FWHT is applied to extract discriminating features from EEG signals, with entropy-based features (ApEn, SampEn, PermEn, FuzzyEn, LogEn) fed into an ANN classifier for focal *vs*. non-focal classification. Feature extraction: non-linear entropy features (ApEn, SampEn, PermEn, FuzzyEn, LogEn) computed from decomposed EEG signals using FWHT. CV: 10-fold CV is employed to evaluate the classifier’s performance. LA: classifies focal and non-focal EEG signals based on entropy measures	University of Bonn (UOB) dataset: Accuracy: 92.80% Sensitivity: 91% Specificity: 94.60% Bern–Barcelona (BB) dataset: Accuracy: 99.50% Sensitivity: 99.70% Specificity: 99.36% Feature performance: Best feature combination: five-feature combination (SampEn, FuzzEn, LogEn, PermEn, and ApEn) UOB dataset: maximum accuracy of 92.80% with a five-feature combination BB dataset: maximum accuracy of 99.50% with a five-feature combination Individual feature performance (BB dataset): SampEn alone: accuracy of 96.84%, sensitivity of 100%, specificity of 93.69% Four-feature combination performance (BB dataset): Combination of SampEn, FuzzyEn, PermEn, LogEn: accuracy of 94.43% Statistical significance: all extracted features were found to be statistically significant (*p* < 0.01) using Student’s *t*-test This study focused on classifying non-focal and focal EEG signals using various entropy measures and an ANN classifier. The results show high accuracy, sensitivity, and specificity, particularly for the BB dataset, indicating strong performance in distinguishing between non-focal and focal EEG signals in epilepsy patients.
Wang Z et al., 2020 (52)	CNN is employed to classify the phase-amplitude cross-frequency coupling (CFC) patterns in SEEG data. Modulation index (MI) comodulograms, representing the coupling between high-frequency amplitude and low-frequency phase, serve as input features to the CNN for distinguishing epileptogenic from normal brain tissue. Feature extraction: MI comodulograms are computed using SEEG signals filtered at two frequency ranges: 1–10 Hz (low frequency) and 30–160 Hz (high frequency). These comodulograms capture two-dimensional CFC patterns across seizures and are directly used for classification. CV: a leave-one-out CV method is used to evaluate the classifier’s performance. The model is trained on five patients and tested on the remaining one, iterating this process for all six patients. LA: the CNN is used to classify SEEG channels as pathological or normal based on the CFC patterns, helping localize the epileptogenic zone.	Proposed pipeline (CNN using MI comodulogram): Average AUC: 0.88 Sensitivity: 0.81 Specificity: 0.79 Traditional CFC strength-based classification method (CNN using only MI strength): Average AUC: 0.67 Sensitivity: 0.61 Specificity: 0.66 Comparison of network architectures: Five fully connected layers (proposed method): Average AUC: 0.88 Single fully connected layer: Average AUC: 0.54 (poor convergence compared to the five-layer network) This study compared the performance of a CNN using MI comodulogram against a traditional CFC strength-based classification method. The proposed pipeline using MI comodulogram significantly outperformed the traditional method in terms of AUC, sensitivity, and specificity. Additionally, the study demonstrated that a network with five fully connected layers performed substantially better than a single fully connected layer.
Sumsky SL et al., 2019 (53)	SVM classifier for the automated identification of the SOZ. The classifier is based on channel ranking derived from ripple-like HFO events in multichannel iEEG recordings. The HFO rates are used to build a time-varying epileptic susceptibility index (SI) for each channel. Feature extraction: the HFO rate is calculated from ripple events detected in consecutive, non-overlapping 10-min epochs. The channels are ranked based on their HFO rates, and the rank values are used to compute an epileptic SI. CV: a K-fold CV approach is used to evaluate the system’s performance. The classifier is trained and validated on different sets of patients, ensuring that training and testing occur on separate groups. LA: the SVM classifier assigns channels to the SOZ based on the epileptic SI.	HFO detection: Average HFO rate in resected volume (RV) channels: 0.88 ± 0.49 events/min Average HFO rate in non-SOZ channels: 0.56 ± 0.24 events/min Significant difference between RV and non-SOZ channels at the group level (*p* = 0.03 for all patients, *p* = 0.01 for class I patients). SI: Average value for RV channels: 0.08 ± 0.02 Average value for non-SOZ channels: 0.03 ± 0.02 Significant difference between RV and non-SOZ channels (*p* < 0.001) Rank-based SVM (proposed method): Average AUC: 0.94 (range: 0.86–0.94 for class I patients, 0.71–0.90 for class >I patients) Significantly outperformed rate-based SVM and rank-based LR (*p* < 0.005) Rate-based SVM: Performed at chance level consistently across all patients Rank-based LR: Performed better than rate-based SVM but worse than rank-based SVM Window-based analysis (30-min windows): Rank-based SVM: average AUC of 0.94, consistently above 0.90 Rate-based SVM: performed close to chance level (AUC = 0.5) Rank-based LR: performed above chance but significantly lower than Rank-based SVM Prediction error: Class I patients: 6.5 ± 1.1% Class >I patients: 12.8 ± 1.2% Significant difference between class I and class >I patients (*p* < 0.0007) Prediction accuracy (class I patients): Exact RV prediction: 46% of cases Underestimation by ~1 electrode: 51% of cases Overestimation or shift: 3% of cases. Prediction accuracy (class >I patients): Larger than RV: 20% of cases (average score: (0, +1.2)) Shifted compared to RV: 79% of cases (average score: (−1.0, +1.4)) Average of 2.4 mislabeled channels per patient Rank-based SVM-All (trained on both class I and class >I patients): Poor overlapping with RV Average error: 2–4 electrodes (corresponding to 3 to 7 cm^2^) No significant difference between class I and class >I patients. Comparison to other methods: Significantly outperformed rate-based SVM and rank-based LR (*p* < 0.005).
Varatharajah Y et al., 2018 (54)	An SVM classifier is employed to predict SOZ using multiple interictal biomarkers such as HFOs, interictal epileptiform discharges (IEDs), and phase-amplitude coupling (PAC). The classifier combines these features to address inter-patient variability and capture the temporal dynamics of epileptic activity. Feature extraction: the biomarkers (HFO, IED, PAC) are extracted from 2-h-long interictal iEEG recordings. Each 2-h recording is divided into non-overlapping 3-s epochs, during which the presence of biomarkers is detected. The results are used to compute local biomarker rates over 10-min windows, reducing the number of features for classification. CV: a 10-fold CV and a leave-one-out approach are used to evaluate model performance. In both methods, distinct training and testing sets ensure that the model is validated on separate patients. LA: the SVM classifier with an RBF kernel assigns electrodes to the SOZ based on the local biomarker rates.	SVM with RBF kernel (non-linear) AUC: 0.79 SVM with linear kernel AUC: 0.57 Non-linear classification boundary between SOZ and non-SOZ electrodes Supervised *vs*. unsupervised approach: Supervised (SVM with RBF kernel) AUC: 0.79 Unsupervised (biomarker incidence rate-based) AUC: 0.56–0.62 (17–23% lower) Biomarker performance (10-fold CV): All biomarkers combined AUC: 0.79 PAC biomarker alone AUC: 0.74 HFO and IED biomarkers alone: not explicitly stated, but lower than PAC CV approaches: 10-fold CV AUC (all biomarkers): 0.79 Leave-1-out CV AUC (all biomarkers): 0.73 Inter-patient variability: Combined biomarkers improved or maintained AUC for >65% of patients compared to individual biomarkers AUC distribution across patients skewed toward higher values when using combined biomarkers Recording duration analysis: 90–120 min recordings showed no statistically significant difference in AUC Suggests 90–120 min may be sufficient for interictal SOZ identification Other metrics: Specificity: ~75% (based on FPR of 25% used for threshold selection) Sensitivity, precision, F1 score: not explicitly stated, but can be inferred from AUC values
Elahian B et al., 2017 (55)	LR classifier utilized for identifying SOZ from electrocorticographic (ECoG) recordings, the classifier leverages features extracted from phase-locking value (PLV), which measures phase synchronization between high gamma activity (80–150 Hz) and lower frequency rhythms (4–30 Hz). This approach aims to differentiate SOZ electrodes from non-SOZ electrodes. Feature extraction: five features are extracted from the PLV values during specific time windows before and after seizure onset. These include PLV positive (a threshold-exceeding feature), duration of PLV positive, PLV peak, PLV mean, and PLV power. These features are calculated from ECoG data acquired in 3-s epochs across both pre-ictal and ictal periods. CV: a 10-fold CV method with L1 regularization is applied to train and validate the LR classifier on data from seizure-free patients. LA: the classifier assigns electrodes to the SOZ based on the PLV features.	Seizure-free patients (6 patients): Total algorithm-positive SOZ (aSOZ) electrodes identified: 54 aSOZ electrodes within resected area: 52 (96%) FPR: 3.7% (2 out of 54 electrodes) 5 out of 6 patients: 100% aSOZ electrodes within the resected area 1 patient: 6 out of 8 aSOZ electrodes within the resected area Comparison with visually identified SOZ (vSOZ) in seizure-free patients: Total vSOZ electrodes: 47 vSOZ electrodes detected as aSOZ: 28 (59.6%) Performance metrics for seizure-free patients: AUC: 69% Accuracy: 83% Precision: 90% Non-seizure-free patients (4 patients): Total aSOZ electrodes identified: 62 aSOZ electrodes within resected area: 43 (69%) aSOZ electrodes outside resected area: 19 (31%) Comparison with vSOZ in non-seizure-free patients: Total vSOZ electrodes: 40 vSOZ electrodes detected as aSOZ: 20 (50%) Additional findings for non-seizure-free patients: 9 aSOZ electrodes were outside the resected area and not visually identified as SOZ Correlation observed between the number of non-resected aSOZ electrodes and poorer seizure outcomes Overall performance: Algorithm demonstrated high precision in identifying SOZ electrodes within resected areas for seizure-free patients Lower accuracy in non-seizure-free patients, but identified potential missed SOZ areas
Dian JA et al., 2015 (56)	An SVM classifier is applied to identify brain regions of interest (ROIs) for epilepsy surgery. The model leverages features derived from both low-frequency oscillations (LFO, 1–80 Hz) and (HFO, 80–400 Hz) in iEEG recordings. It aims to differentiate SOZ channels from non-SOZ channels to guide surgical resection. Feature extraction: using empirical mode decomposition, the iEEG signals are decomposed into intrinsic mode functions (IMFs), capturing the rhythmic components of the data. From these IMFs, time-domain features such as power, RMS power, mean, variance, skewness, and kurtosis are computed for each channel. These features create a unique vector for each channel, representing both LFO and HFO components. CV: a 5-fold CV grid search is performed to tune the SVM’s hyperparameters (penalty parameter 𝐶 and RBF kernel parameter *γ*). The classifier is trained only on channels from the patient with an Engel class I surgical outcome, aiming for high accuracy in identifying SOZ channels. LA: the trained SVM classifier is tested on channels from seizures not used in the training phase. The classifier identifies ROIs (i.e., channels for surgical resection) by combining LFO and HFO features.	Classification accuracies > 95% for all models (LFO, HFO, LFO + HFO) Patients with Engel class I to IV outcomes Classifier performance by feature type: (a) LFO-based classifier: High sensitivity but low specificity Identified many channels outside the resection area (b) HFO-based classifier: Similar pattern to LFO-based classifier Identified many channels outside the resection area (c) Combined LFO + HFO classifier: Improved sensitivity and specificity compared to individual classifiers Best performance in identifying channels within the resection area Patient-specific results: (a) Patient A (Engel class I—seizure-free): LFO + HFO classifier identified only channels within the resection area (b) Patient B (Engel class III—worthwhile improvement): LFO + HFO classifier identified channels within and outside the resection area Suggests ROI larger than the resected area (c) Patient C (Engel class IV—no worthwhile improvement): Classifier did not highlight channels in the resection region Identified channels outside the resection area Suggests incorrect or incomplete SOZ identification Overall performance: Combined LFO + HFO classifier showed the best performance in localizing the SOZ Classifier results correlated with surgical outcomes Potential to identify areas for further resection in non-seizure-free patients Note: Specific quantitative metrics (e.g., sensitivity, specificity, AUC) are not provided for the test data. The results are primarily described qualitatively in relation to the resection areas and surgical outcomes.
Zhu G et al., 2015 (57)	Multiscale K-means (MSK-means) classifier is applied to classify epileptic EEG signals and localize the SOZ using delay permutation entropy (DPE) features. The MSK-means algorithm is designed to improve the performance and efficiency of traditional K-means clustering, particularly for large EEG datasets. Feature extraction: DPE is used to capture the non-linear dynamics of the EEG signals. The optimal delay factor is determined by comparing DPE values across various time delays (lags). The extracted DPE features are fed into the MSK-means classifier, effectively distinguishing between epileptic and non-epileptic signals. LA: the classifier is tested on EEG datasets from epileptogenic and non-epileptogenic brain regions.	DPE index analysis: Evaluated on epileptogenic and non-epileptogenic iEEG zones *τ* (delay lag) range: 1 to 50 Significant difference in DPE indices between epileptogenic and non-epileptogenic zones for τ range 5–30 (p = 0.01) Classification performance: (a) Dataset #50: 100 × 50 dimensional DPE features (b) Dataset #750: 4,500 × 50 dimensional DPE features Best classification results: Maximum accuracy: 93% (Dataset #750) Optimal τ: 18 Best classifier: MSK-means (*κ* = 8) Classifier comparison: MSK-means outperformed SVM and K-means for τ between 6 and 30 Confusion matrix results: (a) SVM with PE (τ = 1): Accuracy: 59% (b) MSK-means with DPE (τ = 18): Accuracy: 93% Improvement over previous methods: 34% higher accuracy compared to PE with SVM Higher than previous work (84% accuracy with SVM on 50 recordings) More robust results with 4,500 test recordings Exceeds existing recorded results (50–80% accuracy) in the literature

ML, machine learning; EC, effective connectivity; SVM, support vector machine; RBF, radial basis function; MLP, multilayer perceptron(s); DI, directed information; MI-GCI, Mutual Information-Guided Granger Causality Index; FD-CCM, frequency-domain convergent cross-mapping; SOZ, seizure-onset zone; AUC, area under the receiver operating curve; DL, deep learning; DeepSIF, deep learning-based source imaging framework; GDeepSIF, generic DeepSIF; PDeepSIF, personalized DeepSIF; CR, concordance rate; LE, localization error; SD, spatial dispersion; CNN, convolutional neural network; SOZ, seizure-onset zone(s); SEEG, stereoelectroencephalography; YI, Youden Index; TLE, temporal lobe epilepsy; FPR, false-positive rate; iEEG, intracranial electroencephelogram; SMOTE, synthetic minority over-sampling technique; ADASYN, adaptive synthetic sampling; msResNet, multiscale residual neural network; KNN, k-nearest neighbors; LR, logistic regression; CV, cross-validation; MEG, magnetoencephalography; STPE, short-time permutation entropy; GSTPE, gradient of STPE; STE, short-term energy; STM, short-time mean; LA, localization approach; BLSTM, bidirectional long short-term memory; LOPO-CV, leave-one-patient-out CV; UWM, University of Wisconsin-Madison; HFO, high-frequency oscillation; RMS, root mean square; aHFA, anomalous high-frequency activity; cHFO, conventional HFO; FWHT, Fast Walsh–Hadamard transform; ANN, artificial neural network; UOB, University of Bonn; BB, Bern–Barcelona; CFC, cross-frequency coupling; MI, modulation index; SI, susceptibility index; RV, resected volume; IED, interictal epileptiform discharges; PAC, phase-amplitude coupling; ECoG, electrocorticography; PLV, phase-locking value; aSOZ, algorithm-positive SOZ; vSOZ, visually identified SOZ; ROI, region of interest; LFO, low-frequency oscillation; IMF, intrinsic mode function; MSK-means, multiscale K-means; DPE, delay permutation entropy.

#### 3.4.1 Traditional machine learning methods

SVMs were widely applied and showed strong performance (43, 53, 54, 56). Root mean square (RMS) and adaptive discriminant analysis (ADA) algorithms were the primary focus of the study by Charupanit et al. (50). An LR with phase-locking value (PLV) properties was used by Elahian et al. (55).

#### 3.4.2 Neural network-based approaches

A modified multiscale-1D-ResNet CNN was utilized by Johnson et al. to analyze the SEEG data (45). Zhao et al. investigated one-dimensional CNNs with specific loss functions [class-balanced (CB) focal loss, cross-entropy (CE)], and various data augmentation methods [EEGAug, synthetic minority over-sampling technique (SMOTE), adaptive synthetic (ADASYN)] (46). Prasanna et al. incorporated entropy measurements and the fast Walsh–Hadamard transform (FWHT) approach to train an artificial neural network (ANN) classifier (51). Cross-frequency coupling (CFC) and mutual information (MI) comodulograms were implemented in the design of the CNN by Wang et al. (52).

#### 3.4.3 Advanced and hybrid models

Effective connectivity (EC) metrics were utilized by Balaji et al. to integrate SVM with graph feature-based models and multilayer perceptrons (MLPs) (43). With both generic and personalized variants, Sun et al. introduced the DeepSIF framework, which leveraged deep neural networks trained on neural mass models (44). The RUSBoost algorithm with varied characteristics, including short-term prediction error (STPE), generalized STPE (GSTPE), and short-term mean (STM), was employed by Bhanot et al. (48).

#### 3.4.4 Comparative studies

Several algorithms, including multiscale residual neural network (msResNet), ResNet, CNN, MLP, k-nearest neighbors (KNN), RF, and LR, were compared by Jeong et al. (47). SZTrack (a CNN–RNN hybrid), CNN–bidirectional long short-term memory (BLSTM), temporal graph convolutional network (TGCN), deep graph convolutional network (GCN), shallow GCN, Wei-CNN, and CNN-2D9 were among the DL models that Craley et al. presented a thorough comparison of (49). Zhu et al. conducted a comparative study of K-means, SVM, and multiscale K-means (MSK-means) algorithms with directed phase envelope (DPE) (57).

### 3.5 Prediction performance

#### 3.5.1 Traditional machine learning methods

Traditional ML methods achieved strong but variable results (Table 2). High sensitivity was observed by Charupanit et al. for their classification methods. Using the receiver operating curve (ROC) analysis, the anomalous high-frequency activity (aHFA) amplitude demonstrated a mean sensitivity of 93.7% for individual subject categorization, whereas the conventional high-frequency oscillation (cHFO) amplitude attained a slightly higher mean sensitivity of 94.1% (50). The cHFO rate metric mean sensitivity was 84.9%, which was still remarkable. Impressive area under the curve (AUC) values were reported by the study for both group-level and individual subject classifications. Individual subject classification yielded mean AUCs of 0.960 (aHFA amplitude), 0.948 (cHFO amplitude), and 0.905 (cHFO rate). Group-level, channel-based ROC analysis showed similar AUC values: 0.956 (cHFO rate), 0.952 (aHFA amplitude), and 0.911 (cHFO amplitude). These high AUC values indicated strong discriminative potential in differentiating between non-SOZ and SOZ channels, especially for amplitude-based measures (50). Varatharajah et al. reported their combined biomarker approach to have a specificity of approximately 75%, based on a threshold selection false-positive rate (FPR) of 25% (54). The non-linear SVM model with the radial basis function (RBF) kernel yielded an AUC of 0.79, far surpassing both the linear SVM (AUC: 0.57) and unsupervised techniques (AUC: 0.56–0.62). Under a 10-fold cross-validation (CV), the combined biomarker method maintained this AUC of 0.79, while the phase-amplitude coupling biomarker alone achieved an AUC of 0.74. Interestingly, compared to individual biomarkers, the combined biomarkers increased or maintained AUC for over 65% of patients, indicating the consistency of this strategy across a range of patient populations (54).

Additionally, a rank-based SVM algorithm for SOZ prediction was proposed by Sumsky et al. The proposed approach achieved a remarkable AUC of 0.94 across all patients (53). Class I patients performed especially well, with AUC values ranging from 0.86 to 0.94, whereas class >I patients reached AUC values between 0.71 and 0.90. Notably, both the rate-based SVM and the rank-based LR models were considerably outperformed by the rank-based SVM (*p* < 0.005) (53). In window-based analysis, the approach performed well, consistently maintaining an average AUC of 0.94 for 30-min windows, consistently above 0.90.

When applied to seizure-free patients, Elahian et al. found that their model had an overall accuracy of 83% (55). Additionally, they reported that their approach had a high sensitivity for locating SOZ electrodes in the resected area for patients who were seizure-free. With a low FPR of 3.7, 96% (52 out of 54) of the algorithmically determined SOZ (aSOZ) electrodes for these patients were located inside the resected region. Additionally, in seizure-free individuals, the algorithm demonstrated a 59.6% sensitivity in identifying visually recognized SOZ (vSOZ) electrodes as aSOZ (55). Dian et al. reported the combined low-frequency oscillation (LFO) + high-frequency oscillation (HFO) classifier outperforming the individual LFO- and HFO-based classifiers in terms of sensitivity and specificity (56).

By contrast, Zhu et al. employed an MSK-means classifier in conjunction with their directed phase lag entropy (DPLE) measure to attain even greater accuracy (57). Their best classification performance values, utilizing a large dataset of 4,500 × 50 dimensional DPLE characteristics, demonstrated a maximum accuracy of 93% (57).

#### 3.5.2 Neural network-based approaches

Neural networks, particularly DL models, have yielded promising results (Table 2). These techniques, which range from CNNs to more intricate structures such as ResNet, have shown encouraging outcomes. Johnson et al. reported that their DL model classified SOZ with a sensitivity of 78.1% (95% CI 67.8–88.4%) and a specificity of 74.6% (95% CI 68.7–80.5%) (45). A Youden Index (YI) of 52.7 (95% CI 43.7–61.8) was determined by the study, showing a performance that was balanced between sensitivity and specificity. This index did not vary throughout the course of the various time periods; for example, the YI for the 0–350 ms window was 52.5 (95% CI 42.1–62.9). With a 91.5% (95% CI 89.7–93.3%) sensitivity for ipsilateral SOZ prediction, the model exhibited especially high sensitivity for unilateral mesial temporal lobe epilepsy. Performance differed for various forms of epilepsy: neocortical temporal SOZs achieved a sensitivity of 64.4%, whereas bilateral mesial temporal lobe epilepsy showed sensitivities of 68.9 and 67.9% for left and right mesial temporal SOZs, respectively (45).

A variety of data augmentation techniques were investigated by Zhao et al. reporting a range of patient performance indicators (46). Their accuracy ranged roughly from 85 to 99% and demonstrated the potential of data augmentation techniques in improving model performance. The data augmentation techniques (EEGAug, SMOTE, ADASYN) yielded a broad range of recall values across the patients, falling in the range of 30–90% (46). Although sensitivity significantly varied among individuals, specificity remained consistently high, ranging from 90 to 99%. The observed variation in sensitivity and accuracy among patients implies that the efficacy of these techniques might depend on the unique attributes of each patient or the quality of the data (46).

The results for the msResNet model, which demonstrated remarkable accuracy in both the model and validation cohorts, were reported by Jeong et al. (47). Using 5-fold CV, the model cohort achieved 96 and 99% accuracy on the test set and training set, respectively. In that sample, the model exhibited high sensitivity, scoring 92% for lesional MRI patients and 91% for non-lesional MRI patients. The accuracy was 94% for non-lesional MRI patients and 97% for patients with lesional MRI (47). The performance was reduced in the validation group, with a balanced accuracy of 75% for patients with lesional MRI and 67% for non-lesional MRI patients. However, in this sample, sensitivity dropped to 56% for non-lesional MRI patients and 64% for lesional MRI patients, suggesting a potential challenge in generalizability (47). Additionally, the msResNet model exhibited excellent specificity for both MRI modalities and cohorts. Specificity in the model cohort was very good, with values of 98% for lesional MRI patients and 96% for non-lesional MRI patients. The specificity remained robust at 85% for lesional MRI patients and 78% for non-lesional MRI patients even in the validation sample, where overall performance declined, demonstrating the model’s high capacity to accurately detect non-SOZ areas (47). Additionally, the study provided AUC values for their suggested MRI marker (μi) in conjunction with iEEG signals (47). This combination increased AUC from 0.56–0.62 (iEEG alone) to 0.64–0.65 (non-lesional MRI group), which represents a 5–15% improvement. Nevertheless, the combination did not show improvement in the lesional MRI group; the AUC for both iEEG + μi.7 and iEEG alone remained at 0.70–0.77 (47). Furthermore, it was reported that the msResNet model outperformed MLP, CNN, and ResNet18, as well as other ML approaches, with gains in overall accuracy of up to 47% for lesional MRI instances and up to 16% for non-lesional MRI cases (47). Moreover, Prasanna et al. demonstrated good accuracy using an ANN classifier with features based on entropy (51). They obtained an accuracy of 92.80% on the University of Bonn (UOB) dataset and 99.50% on the Bern–Barcelona (BB) dataset. Sensitivity values of 91% for the UOB dataset and 99.7% for the BB dataset were reported. High specificity values of 94.6 and 99.36% for the corresponding datasets accompanied these results (51). Various entropy measurements were combined into five features to obtain the optimum performance. These findings indicate that entropy-based characteristics may be used with CNNs to achieve precise SOZ prediction (51).

Further exploring the potential of neural networks, Wang et al. suggested a CNN pipeline for SOZ prediction that employed the MI comodulogram (52). Their method provided 79% specificity and 81% sensitivity. The study reported that the suggested CNN pipeline exhibited an average AUC of 0.88, which was superior to the average AUC of 0.67 obtained by the standard CFC strength-based classification technique, which had lower sensitivity (61%) and specificity (66%) (52). The five-layer fully connected network (AUC: 0.88) substantially outperformed a single-layer network (AUC: 0.54), further demonstrating the significance of network architecture in the study. These findings demonstrate how cutting-edge CNN architectures and novel feature representations may enhance the precision of SOZ prediction (52).

#### 3.5.3 Advanced and hybrid models

The field of SOZ prediction has advanced significantly as a result of the development of complex and hybrid models (Table 2). These innovative methods integrated methodologies from ML, signal processing, and information theory. By leveraging approaches including MI quantification, directed information (DI) analysis, and frequency-domain feature extraction, these models offered enhanced capabilities for interpreting the intricate patterns of epileptic activity. Furthermore, the development of customized DL architectures represents a significant advance toward patient-specific analysis. It has been found that advanced and hybrid models, which include MI, DI, and frequency-domain characteristics, perform exceptionally well in tasks related to localizing seizures. High-performance metrics were reported by Balaji et al. for their three EC measure-based approaches (43). Using an MLP classifier with 10% sparsity, the DI-based features obtained 92.12% accuracy, with 85.3% sensitivity and 92.8% specificity. Using an SVM with an RBF kernel at 90% sparsity, the Mutual Information-Guided Granger Causality Index (MI-GCI)-based features demonstrated the greatest specificity of 96.32% and the highest sensitivity of 89.74%. The maximum accuracy was reported at 95.72% (43). With an accuracy of 94.10% using an SVM with an RBF kernel at 10% sparsity, the frequency-domain convergent cross-mapping (FD-CCM)-based features also performed well; they reached a sensitivity of 92.3% and a specificity of 94.25%. The study notably revealed remarkable AUC values for each of the three measures: the AUC for features based on DI was 0.89, that of MI-GCI-based features was 0.9, and that of FD-CCM-based features was the highest at 0.93 (43).

Further advancing the field of hybrid models, Sun et al. presented a customized DL method for SOZ prediction (44). When compared to the generic DeepSIF (GDeepSIF), their personalized DeepSIF (PDeepSIF) model exhibited better sensitivity metrics. With a sublobar sensitivity of 77% and a specificity of 99%, PDeepSIF outperformed GDeepSIF, which achieved only a sublobar sensitivity of 66% and a specificity of 97%. Alongside this sensitivity improvement, there was a notable rise in the sublobar concordance rate, which increased from 83 to 93% (44). Adopting PDeepSIF was reported to significantly reduce both localization error (LE) and spatial dispersion (SD). PDeepSIF achieved 15.78 ± 5.53 mm for LE, whereas GDeepSIF achieved 24.86 ± 10.40 mm. With an SD of 8.19 ± 8.14 mm, PDeepSIF significantly outperformed GDeepSIF, which had a score of 21.90 ± 19.03 mm (44). Furthermore, PDeepSIF outperformed traditional methods such as LCMV, sLORETA, and CMEM in both metrics. Conventional techniques yielded LE of 25.94 ± 7.07 mm (LCMV), 22.89 ± 6.14 mm (sLORETA), and 31.35 ± 15.86 mm (CMEM). Moreover, conventional techniques showed much higher SD values than PDeepSIF: 33.27 ± 16.19 mm (LCMV), 29.10 ± 14.66 mm (sLORETA), and 29.15 ± 18.66 mm (CMEM) (44). These findings, which exhibited better accuracy and precision than both generic models and traditional techniques, demonstrate the promise of individualized DL approaches in improving SOZ prediction performance.

Similarly, Bhanot et al. achieved remarkable findings, obtaining 93.4% accuracy in whole-head SOZ prediction (48). Their model exhibited 93% specificity and 93% sensitivity. The study produced outstanding AUC values, demonstrating significant discriminative ability of the algorithm even with strongly skewed data, with an AUC of 0.97 for whole-head SOZ prediction and a minimum AUC of 0.95 across all areas for epileptogenic lobe localization (48). Along with the high AUC scores, these balanced sensitivity and specificity values indicated robust performance in differentiating between the ictal and interictal phases. Furthermore, they demonstrated high accuracy in region-specific classifications of epileptogenic lobe localization, underscoring the potential of sophisticated models for accurate SOZ prediction. Moreover, Craley et al. evaluated their SZTrack model on two datasets (49). SZTrack obtained a sensitivity of 83.5% at the seizure level for SOZ prediction on the JHH dataset, which was marginally less than the best-performing baseline (CNN-BLSTM) at 91.9%. The sensitivity for SOZ prediction dropped to 63.9% on the University of Wisconsin-Madison (UWM) dataset, which was used to assess generalization, although it was still higher than the CNN-BLSTM baseline (52.3%) (49). For lateralization, SZTrack outperformed other baselines with a maximum average accuracy of 82.6%, and it achieved an average accuracy of 58.7% in lobe classification, which was slightly lower than 60.5% for the top-performing TGCN. Notably, in 58.8% of patients in the JHH dataset, the model correctly recognized both the hemisphere and the lobe; in the remaining cases, the model correctly identified either the hemisphere or the lobe (49). With promising generalization capabilities, the results demonstrated the potential of SZTrack in SOZ prediction.

## 4 Discussion

### 4.1 Overview of machine learning approaches for seizure-onset zone prediction

This systematic review evaluated 15 studies investigating ML approaches for SOZ prediction in patients with DRE. The findings demonstrated considerable heterogeneity in methodological approaches and performance metrics. The wide variety of methods illustrated both the complexity of the problem and the continuous quest for the best prediction models in this field. Although the usage of classical approaches continued to demonstrate their usefulness in some circumstances, the trend toward DL and hybrid models indicates a move toward capturing more complex patterns in the data. Traditional ML methods, particularly SVM-based approaches, exhibited consistent performance with AUC values ranging from 0.79 to 0.94 (Table 2). Among these, rank-based methods demonstrated particular promise. Sumsky et al. reported that their rank-based SVM algorithm achieved superior AUC values, especially for class I patients (53). These findings suggest that rank-based methods have the potential to increase the accuracy of SOZ prediction. This insight is particularly valuable, as it may help in developing more refined models for patients with a higher likelihood of surgical success (58).

However, it is important to note that although traditional ML methods have shown good performance, the trend in recent years has been toward more complex models. Neural network-based techniques, especially DL models such as CNNs and ResNets, have emerged as effective tools, frequently surpassing conventional approaches (59). High AUC, sensitivity, and accuracy scores have been demonstrated by these models; some studies have reported performance values for these measures that are higher than 0.9 (Table 2). The resilience of DL models has been shown in their notable consistency in performance across various types of epilepsy and temporal scales (60). Furthermore, advanced and hybrid models that integrate concepts from information theory, signal processing, and ML have demonstrated significant promise (61). Methods incorporating MI, DI, and frequency-domain characteristics have shown high values for AUC, sensitivity, and accuracy, frequently above 0.9 (Table 2).

Moreover, studies have also shown that individualized DL architectures are a promising approach, demonstrating notable improvements in SD, accuracy, and localization errors compared with generic models and conventional approaches (Table 2). The evidence as a whole indicates that advanced neural network-based techniques and hybrid models that incorporate novel feature representations and patient-specific architectures hold the greatest promise for precise and reliable SOZ prediction in patients with DRE, even though reported performance varied across studies and methodologies. Notably, personalized approaches, such as PDeepSIF, demonstrated marked improvements over generic models, particularly in reducing localization error and spatial dispersion (Table 2).

### 4.2 Comparative performance analysis of algorithmic modeling approaches

The high-performance metrics reported by Balaji et al. for their EC measure-based approaches are particularly noteworthy (43). These methods demonstrated strong discriminative power in differentiating between SOZ and non-SOZ electrodes. The combination of high AUC values with good sensitivity and specificity metrics suggests that EC-based features may be particularly effective for SOZ prediction. This finding underscores the potential of incorporating connectivity measures into ML models for epilepsy, as they may capture important network-level information relevant to seizure onset (62). However, it is important to note that these results were obtained from a specific dataset and context, and further validation in diverse patient populations and epilepsy types is necessary to establish the generalizability of these approaches.

The comparative analysis of ML approaches revealed several important patterns. DL architectures generally outperformed traditional ML methods, particularly in handling complex, multimodal data (Table 2). However, this superior performance often came at the cost of increased computational complexity and reduced interpretability (63). Performance metrics varied significantly across studies, with sensitivity varying from 56 to 99.7% and specificity from 74.6 to 99.36% (Table 2). This variability could be potentially influenced by clinical heterogeneity driven by several factors, including but not limited to dataset size, data acquisition methods, feature selection approaches, and patient characteristics (64). Notably, studies utilizing SEEG data typically achieved higher accuracy than those using standard EEG, suggesting the importance of data quality and spatial resolution (65).

### 4.3 Clinical translation challenges

Our findings both reinforced and expanded upon the findings of the previous reviews in this field. Although earlier studies have demonstrated the potential of ML in epilepsy management (39, 66), our review specifically highlighted the evolution toward more sophisticated, hybrid approaches that combine multiple data modalities and learning architectures. The superior performance of personalized models, particularly in reducing localization error, represents a novel insight not previously emphasized in the literature. However, our findings regarding the impact of data quality and quantity on model performance align with previous observations in the context of medical ML applications (67).

These findings have substantial clinical implications but face implementation challenges. Despite promising accuracy improvements, integrating ML approaches into clinical workflows requires careful consideration of several factors. The requirement for high-quality, standardized data acquisition poses logistical and resource challenges. Moreover, dataset imbalance represents a significant technical hurdle (68), as SOZ regions typically constitute a small fraction of recorded channels and brain volume, potentially biasing algorithms toward high specificity at the expense of sensitivity. Furthermore, the interpretability of complex ML models, particularly deep neural networks, remains limited, creating a “black box” problem that may reduce clinician trust and adoption. The need for computational expertise and infrastructure may also limit adoption in clinical settings, particularly in resource-constrained environments. Additionally, the cost-effectiveness of implementing these systems, particularly more complex DL architectures, warrants careful evaluation against contemporary standard practices, considering not only software and hardware requirements but also personnel training, maintenance costs, and potential savings from improved surgical outcomes. Generalizability across diverse healthcare settings with varying resources and expertise also remains a critical concern for widespread clinical implementation.

Furthermore, an important consideration in evaluating the clinical utility of SOZ prediction methods is the long-term postoperative course and seizure recurrence patterns. Although some patients may experience immediate and sustained seizure freedom following surgical intervention, a subgroup of patients may experience seizure recurrence after a variable interval ranging from months to years (69). In such cases, the recurrent seizures may have an identical or similar semiology, suggesting that the initial intervention did not effectively treat the SOZ due to insufficient or inaccurate localization (70). Additionally, some patients may have multiple SOZs, leading to the recurrence of seizures with a different semiology after the initial intervention (71). These observations highlight the importance of accurate and comprehensive SOZ localization. Furthermore, it is important to note that the included studies varied in their length of postoperative follow-up, with 87% (*n* = 13) of studies not specifying the length of follow-up (43, 44, 46–49, 51–57) (Table 1). Given the potential for seizure recurrence over longer periods, studies with longer follow-up intervals, such as Zhao et al. may provide more reliable insights into the long-term clinical utility of the investigated SOZ prediction methods (46).

### 4.4 Methodological considerations and ground truth challenges

The heterogeneity in data acquisition methods across included studies introduces potential biases and complicates direct comparisons of results. This variability, combined with selection biases in patient populations and inconsistent reporting protocols, underscores the urgent need for standardized data collection and processing methods (72). Moreover, the divergence in the definition and validation of outcome measures was recognized as a critical challenge in evaluating ML approaches for SOZ prediction. To address these limitations, we recommend the development of standardized benchmarking protocols, including common performance metrics and validation datasets, to facilitate meaningful comparisons across studies and enhance clinical applicability (73).

A fundamental challenge in evaluating ML approaches for SOZ prediction is the definition and reliability of “ground truth” labels adopted for algorithmic model training and validation. Clinically determined SOZ labels are inherently subject to interobserver variability, limitations in sampling coverage, and interpretative biases. This creates a circular problem: ML models trained on potentially imperfect clinical labels may perpetuate rather than overcome existing limitations in SOZ identification. The ultimate validation metric should, thus, be long-term seizure freedom following surgical resection of the algorithm-identified regions, as this represents the true clinical goal of SOZ prediction. However, only 27% (*n* = 4) of studies explicitly included Engel/ILAE class I patients in their analysis, and 87% (*n* = 13) of studies did not specify follow-up duration. This highlights a critical gap between technical performance metrics and clinically meaningful outcomes. We, therefore, suggest that seizure freedom for at least 2 years should be considered the gold standard for validating ML predictions, as early surgical success may not translate to sustained seizure control (69, 74). Moreover, successful surgical outcomes may result not only from accurate SOZ localization but also from the disconnection of seizure circuits through peri-SOZ white matter resection (75, 76). This distinction is important when evaluating ML performance, as algorithms trained solely on SOZ identification may not capture the full complexity of successful surgical intervention.

### 4.5 Network-level considerations in seizure-onset zone prediction

It should also be noted that the role of neural networks in epilepsy extends beyond the immediate SOZ, involving complex interactions across distributed brain regions that may be crucial for both seizure generation and propagation (77, 78). Epilepsy can lead to widespread alterations in both structural and functional connectivity, affecting multiple brain networks, including the default mode, salience, and attention networks (79, 80). These network-level changes may persist even after successful surgical intervention, highlighting the dynamic nature of epilepsy-related brain plasticity (81). In temporal lobe epilepsy, for instance, studies have demonstrated alterations in hippocampal-cortical networks that extend well beyond the temporal lobe, with implications for cognitive function and surgical outcomes (82, 83). Post-surgical network reorganization has been observed, with some patients showing normalization of previously disrupted networks, while others demonstrate persistent alterations that may contribute to seizure recurrence (81, 84). Understanding these network-level changes is particularly relevant for ML approaches to SOZ prediction, as successful surgical outcomes depend on both the accurate identification of the epileptogenic zone and an understanding of its relationship with broader network dynamics (85). This network perspective is especially crucial in complex epilepsies, where multiple nodes within a network may contribute to seizure generation and propagation (86). Future ML approaches will, therefore, benefit from incorporating these network-level considerations, potentially through the integration of functional connectivity metrics and longitudinal network analysis in their predictive models (87, 88).

### 4.6 Future research directions

Future research directions should address several other key areas. Larger, prospective studies are needed to validate the performance of promising ML approaches, particularly personalized models. The standardization of data acquisition and preprocessing methods would facilitate more meaningful comparisons between approaches. Investigation of model interpretability and validation of ML-derived predictions against surgical outcomes are crucial. Technical developments should focus on improving computational efficiency, reducing the need for extensive preprocessing, and developing more robust approaches for handling limited and ‘noisy’ data. Evaluating multiple ML algorithms on a uniform, well-curated dataset with standardized performance metrics and long-term (≥1–2 years) seizure freedom validation would provide insights into their relative strengths and limitations, helping establish evidence-based guidelines for clinical algorithm selection.

**Figure 3:**
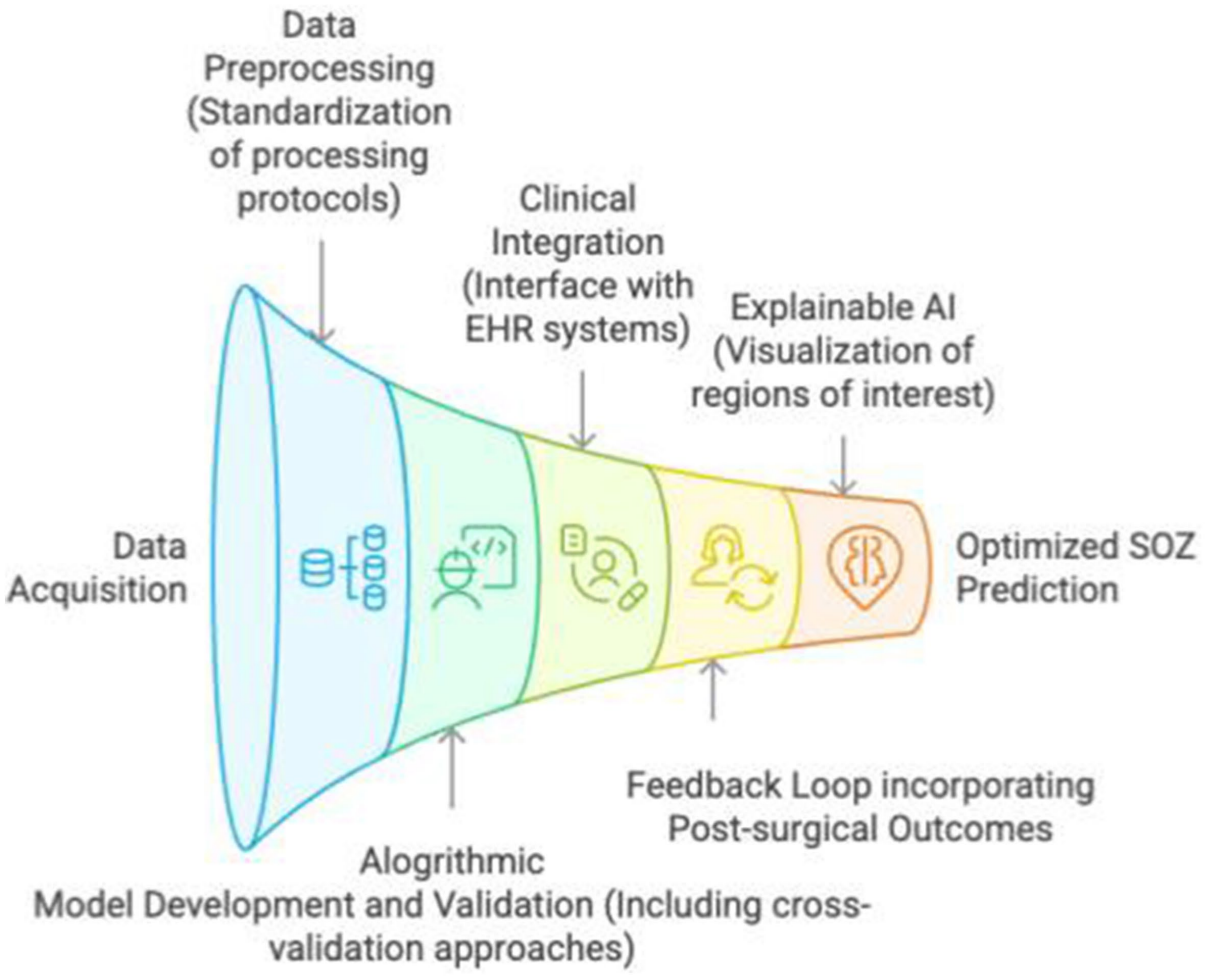
Conceptual framework outlining the pipeline for optimized seizure-onset zone(s) prediction using machine learning in patients with drug-resistant epilepsy.

To facilitate clinical integration, a structured implementation framework is proposed. Prospective multicenter validation studies should include both academic epilepsy centers and community hospitals to reflect diverse clinical environments. Developing explainable artificial intelligence techniques specific to SOZ prediction is also essential for fostering clinician trust, potentially through visualization tools that highlight regions of interest in a clinically interpretable manner. Moreover, practical implementation requires user-friendly software interfaces that seamlessly integrate with existing clinical workflows and electronic health records, presenting ML predictions alongside traditional clinical data (Figure 3). Furthermore, establishing clear regulatory pathways for ML-based decision support tools will ensure appropriate validation standards and facilitate adoption in epilepsy surgery planning.

Future studies should also focus on direct comparisons between ML-enhanced non-invasive methods and current invasive standards of care. If ML approaches can demonstrate comparable or superior accuracy using non-invasive data, this could represent a significant advancement in presurgical evaluation for epilepsy, potentially reducing the need for invasive procedures in some patients. However, until such superiority is conclusively demonstrated, the results of these predominantly EEG-based ML studies should be interpreted cautiously in the context of current clinical practice. Future research should also prioritize the development and validation of ML models specifically designed to assist in SOZ prediction for complex clinical cases with more than one potential epileptogenic zone, as this is where the potential clinical impact could be most significant. EC-based methods should be explored in such complex epilepsy cases and compared to other advanced ML techniques in larger, prospective studies.

Beyond conventional approaches, several innovative directions also warrant exploration. Federated learning represents a promising paradigm that could enable collaborative model development across multiple epilepsy centers without transferring sensitive patient data, addressing both privacy concerns and the challenge of limited dataset sizes at individual institutions. The integration of multimodal data beyond neurophysiological recordings, including genomics, proteomics, and detailed neuropsychological profiles, could enhance algorithmic model performance by capturing and better accounting for the biological mechanisms underlying epileptogenesis. Furthermore, ML approaches could expand beyond SOZ prediction for predicting outcomes stratified for different intervention types such as resection, ablation, and neurostimulation, potentially enabling treatment selection. Moreover, novel self-supervised learning techniques might reduce reliance on labeled data, addressing the ground truth challenges inherent in SOZ determination. In addition to this, the development of adaptive ML systems that incorporate post-surgical outcomes and network reorganization data could lead to continuously improving algorithmic models that account for the dynamic nature of epileptic networks over time.

Advanced multimodal fusion techniques deserve particular attention, focusing on developing sophisticated methods that synchronize and integrate data across temporal and spatial scales, such as combining high-temporal-resolution EEG with high-spatial-resolution imaging. Wearable and minimally invasive devices coupled with ML algorithms represent another promising frontier, potentially enabling continuous monitoring of seizure dynamics in real-world settings and providing unprecedented longitudinal data for model refinement. Patient-specific forecasting models that predict not only SOZ location but also seizure probability and management response could potentially transform epilepsy management by enabling dynamic management adjustments based on individual seizure patterns and neural network states. Additionally, developing ML approaches that distinguish between the SOZ and *symptomatogenic zone* could further provide more comprehensive guidance for surgical planning, potentially improving both seizure control and functional outcomes.

### 4.7 Limitations

This systematic review has several methodological strengths, including comprehensive database searching, rigorous inclusion criteria, and systematic quality assessment using QUADAS-2. However, important limitations must be acknowledged. The heterogeneity in reporting standards and performance metrics complicates direct comparisons between studies. Dataset heterogeneity was also substantial, with variations in patient demographics, epilepsy types, and recording parameters across included studies. The predominance of retrospective designs and relatively small patient cohorts in many studies may have limited the generalizability of the findings. The mean sample size was only 23 patients, with many studies including fewer than 15 subjects, increasing the risk of overfitting and limiting statistical power. Additionally, publication bias favoring positive results cannot be excluded. This bias may be particularly pronounced in ML research, where negative results or underperforming models are substantially less likely to be published. In addition to these, the preponderance of EEG-based studies in this systematic review raises an important question about the selective potential of ML approaches to enhance non-invasive SOZ prediction methods only. Although these non-invasive techniques offer advantages in terms of patient comfort and reduced procedural risks, their ability to match or exceed the precision of intracranial monitoring remains a critical area of investigation (89).

The high adoption rate of EEG in ML studies may reflect its widespread availability and ease of data collection, rather than its superiority for SOZ prediction. Furthermore, a significant limitation identified in our review was the potentially limited clinical utility of many of the studied ML approaches for patients with more complex or difficult-to-diagnose types of epilepsy. Although several studies reported high-performance metrics, these were often in the context of more straightforward cases, such as unilateral mesial temporal lobe epilepsy. For instance, Johnson GW et al. reported high sensitivity (91.5%) for ipsilateral SOZ prediction in unilateral mesial temporal lobe epilepsy, but considerably lower sensitivity for neocortical temporal epilepsy (64.4%) and bilateral mesial temporal lobe epilepsy (68.9 and 67.9% for left and right, respectively) (45). This pattern suggests that current ML approaches may not adequately address the challenges posed by more complex epilepsy types, which are often the cases where accurate SOZ localization is most crucial and challenging in clinical practice.

Furthermore, the scarcity of reported data prevented an analysis from being conducted to explore surgical intervention type, postoperative Engel class outcome subgroups, neurostimulation type, and neurostimulation outcomes as possible confounders to SOZ prediction performance. Moreover, substantial heterogeneity in data collection protocols and limited adoption of non-EEG methods for the collection of data prohibited a comparison of SOZ prediction performance from being made in the context of non-invasive *versus* invasive methods for data collection. Exploration of the probable superiority of SEEG-based data collection over subdural grids and strips, considering the superior spatial coverage of the former, could also not be undertaken.

## 5 Conclusion

This systematic review systematically synthesized the application of ML for SOZ prediction in patients with DRE and demonstrated that ML approaches, particularly DL architectures and personalized models, offer promising solutions for SOZ prediction. Traditional ML methods showed reliable performance, while advanced neural networks achieved superior accuracy in many cases, with some models reaching accuracy rates above 90%. The emergence of hybrid and personalized approaches represents a significant advancement, potentially offering more precise and patient-specific SOZ prediction. These developments have important clinical implications, as improved prediction accuracy could enhance surgical planning and potentially lead to better outcomes in epilepsy surgery. However, successful clinical implementation will require addressing challenges in data standardization, computational infrastructure, and clinical workflow integration. As ML technologies continue to evolve, future developments focusing on larger-scale validation, improved interpretability, and streamlined clinical integration will be crucial in realizing the full potential of these approaches for SOZ prediction in particular and epilepsy care in general.

## Data Availability

The original contributions presented in the study are included in the article/Supplementary material, further inquiries can be directed to the corresponding authors.
