# Supplementary material for: Application of machine learning approaches to predict seizure-onset zones in patients with drug-resistant epilepsy: a systematic review
Source: Front Neurol. 2025 Dec 1;16:1687144. doi: 10.3389/fneur.2025.1687144 (PMC12702746; doi:10.3389/fneur.2025.1687144)
Supplement: Supplementary file 2 [file Data_Sheet_2.pdf]

Supplementary Material

Prospective Study Protocol & Search Strategy Implementation

- **P**atients
  - Adult and pediatric patients (all ages) with drug-resistant epilepsy (DRE) as defined by the International League Against Epilepsy (ILAE)
  - Patients who have undergone pre-surgical evaluation for potential epilepsy surgery
  - Patients with confirmed epilepsy diagnosis based on clinical assessment and EEG findings
  - Both lesional and non-lesional epilepsy cases
    - **Exclusion:**
      - Patients with non-epileptic seizures
      - Patients with provoked seizures (such as secondary to alcohol withdrawal or acute metabolic disturbances)
      - Patients with drug-responsive epilepsy
      - Mixed patient populations where data from patients with DRE could not be separately extracted
- **I**ntervention (**I**ndex Test)
  - Studies applying any machine learning (ML) algorithm for seizure onset zone (SOZ) prediction
  - Both supervised and unsupervised ML approaches
  - Traditional ML methods (such SVM, Random Forest, or k-means)
  - Deep learning architectures (such as CNN, RNN, LSTM, or ResNet)
  - Hybrid and ensemble models
  - Studies using any neurophysiological data type (EEG, SEEG, ECoG, MEG) as input
    - **Exclusion:**
      - Non-ML computational approaches (such as simple statistical methods without learning components)
      - Studies using ML only for seizure detection without SOZ localization
      - Studies using ML for other epilepsy applications (such as drug response prediction or seizure forecasting) without SOZ localization
      - The ML algorithm architecture and results not reported in sufficient detail for assessment of the respective approach
- **C**omparator
  - Studies comparing ML approaches to clinician visual analysis
  - Studies comparing ML approaches to established localization methods (e.g., visual EEG analysis, conventional source localization)
  - Studies comparing different ML algorithms against each other
  - Studies without explicit comparison with or without validation against surgical outcomes or expert consensus

        - **Exclusion:**
            - -

- **O**utcome
    - Primary outcomes
        - Accuracy, sensitivity, and specificity of SOZ localization
        - Area Under the Curve (AUC) values
        - Localization error measurements
        - Spatial dispersion metrics
        - Positive and negative predictive values
        - F1-scores and other performance metrics
    - Secondary outcomes
        - Correlation with post-surgical outcomes (such as Engel or ILAE classification)
        - Computational efficiency metrics
        - Model interpretability assessments
        - Comparison of performance across different epilepsy types
            - **Exclusion:**
                - Studies reporting only qualitative outcomes without quantitative performance metrics
                - Studies without clear definition of performance metrics
                - Studies reporting only technical outcomes without clinical relevance
- **S**tudy design
    - Inclusion:
        - Randomized-controlled trial
        - Cohort study
        - Case-control study
        - Case series with ≥ 3 patients
    - Exclusion:
        - Case series with < 3 patients
        - Case reports
        - Letter to editor, commentaries and all other such types of correspondences
        - Literature reviews

MeSH terms were acquired for aforementioned relevant terms from the MeSH resource of NCBI.

Search strategy implemented on: August 19, 2024

- **PubMed**
    - "epileptic zone"[Title/Abstract] OR "epileptogenic zone"[Title/Abstract] OR "seizure onset zone"[Title/Abstract] OR "seizure zone"[Title/Abstract] OR "zone localization"[Title/Abstract] OR "zone localisation"[Title/Abstract] OR "zone detection"[Title/Abstract] 2,930
    - "Electrocorticography"[MeSH Terms] OR "Electroencephalography"[MeSH Terms] OR "Electroencephalography Phase Synchronization"[MeSH Terms] OR "Magnetoencephalography"[MeSH Terms] 182,926
    - "Machine Learning"[MeSH Terms] OR "Unsupervised Machine Learning"[MeSH Terms] OR "Supervised Machine Learning"[MeSH Terms] OR "Deep Learning"[MeSH Terms] OR "neural networks, computer"[MeSH Terms] 116,123
    - **#1 AND #2 AND #3** **36**
- **CSDR**
    - (epileptic zone):ti,ab,kw 25
    - (epileptogenic zone):ti,ab,kw 30
    - (seizure onset zone):ti,ab,kw 19
    - (seizure zone):ti,ab,kw 38
    - (zone localization):ti,ab,kw 47
    - (zone localisation):ti,ab,kw 47
    - (zone detection):ti,ab,kw 235
    - #1 OR #2 OR #3 OR #4 OR #5 OR #6 OR #7 305
    - MeSH descriptor: [Electrocorticography] explode all trees 17
    - MeSH descriptor: [Electroencephalography] explode all trees 6697
    - MeSH descriptor: [Electroencephalography Phase Synchronization] explode all trees 75
    - MeSH descriptor: [Magnetoencephalography] explode all trees 256
    - #9 OR #10 OR #11 OR #12 6891
    - MeSH descriptor: [Machine Learning] explode all trees 1002
    - MeSH descriptor: [Unsupervised Machine Learning] explode all trees 5
    - MeSH descriptor: [Supervised Machine Learning] explode all trees 82
    - MeSH descriptor: [Deep Learning] explode all trees 328
    - MeSH descriptor: [Neural Networks, Computer] explode all trees 638
    - #14 OR #15 OR #16 OR #17 OR #18 1289
    - **#8 AND #13 AND #19** **ZERO**
- **Epistemonikos**
    - (title:(epileptic zone) OR abstract:(epileptic zone)) OR (title:(epileptogenic zone) OR abstract:(epileptogenic zone)) OR (title:(seizure onset zone) OR abstract:(seizure onset zone)) OR (title:(seizure zone) OR abstract:(seizure zone)) OR (title:(zone localization) OR abstract:(zone localization)) OR (title:(zone localisation) OR abstract:(zone localisation)) OR (title:(zone detection) OR abstract:(zone detection)) 1,313
    - (title:(Electrocorticography) OR abstract:(Electrocorticography)) OR (title:(Electroencephalography) OR abstract:(Electroencephalography)) OR (title:(Magnetoencephalography) OR abstract:(Magnetoencephalography)) 4,208

        - (title:(Machine Learning) OR abstract:(Machine Learning)) OR (title:(Deep Learning) OR abstract:(Deep Learning)) OR (title:(Neural network) OR abstract:(Neural network)) 24,672
        - **#1 AND #2 AND #3** **2**

- Total articles obtained: 38
    - Duplicates removed: ZERO
        - Articles screened: 38
            - **Articles included from search strategy implementation: 15**

- **Articles excluded (n= 23)**
  - **Full-texts inaccessible (n= 2)**
    - Yin N, Han Y, Wang L, Yang F, Li J, Xu G. Localization of epileptogenic zone based on time-varying effective networks. Epilepsy Res. 2024;205:107409. doi:10.1016/j.eplepsyres.2024.107409
    - Chybowski B, Klimes P, Cimbalnik J, et al. Timing matters for accurate identification of the epileptogenic zone. Clin Neurophysiol. 2024;161:1-9. doi:10.1016/j.clinph.2024.01.007
  - **Animal study (n= 1)**
    - Bou Assi E, Nguyen DK, Rihana S, Sawan M. A Functional-Genetic Scheme for Seizure Forecasting in Canine Epilepsy. IEEE Trans Biomed Eng. 2018;65(6):1339-1348. doi:10.1109/TBME.2017.2752081
  - **Different research question (n= 7)**
    - Li Z, Zhao B, Hu W, et al. Machine learning-based classification of physiological and pathological high-frequency oscillations recorded by stereoelectroencephalography. Seizure. 2023;113:58-65. doi:10.1016/j.seizure.2023.11.005
    - Chahid A, Alotaiby TN, Alshebeili S, Laleg-Kirati TM. Feature Generation and Dimensionality Reduction using the Discrete Spectrum of the Schrödinger Operator for Epileptic Spikes Detection. Annu Int Conf IEEE Eng Med Biol Soc. 2019;2019:2373-2376. doi:10.1109/EMBC.2019.8856702
    - Meisel C, Bailey KA. Identifying signal-dependent information about the preictal state: A comparison across ECoG, EEG and EKG using deep learning. EBioMedicine. 2019;45:422-431. doi:10.1016/j.ebiom.2019.07.001
    - Amiri M, Frauscher B, Gotman J. Interictal coupling of HFOs and slow oscillations predicts the seizure-onset pattern in mesiotemporal lobe epilepsy. Epilepsia. 2019;60(6):1160-1170. doi:10.1111/epi.15541
    - Hao Y, Khoo HM, von Ellenrieder N, Zazubovits N, Gotman J. DeepIED: An epileptic discharge detector for EEG-fMRI based on deep learning. Neuroimage Clin. 2017;17:962-975. Published 2017 Dec 5. doi:10.1016/j.nicl.2017.12.005
    - Tomlinson SB, Porter BE, Marsh ED. Interictal network synchrony and local heterogeneity predict epilepsy surgery outcome among pediatric patients. Epilepsia. 2017;58(3):402-411. doi:10.1111/epi.13657
    - Sinha N, Dauwels J, Kaiser M, et al. Predicting neurosurgical outcomes in focal epilepsy patients using computational modelling. Brain. 2017;140(2):319-332. doi:10.1093/brain/aww299
  - **Machine learning not adopted (n= 2)**

- Hospices Civils de Lyon. MEG Versus EEG HR for the Localization of the Epileptogenic Zone as Part of the Pre-Surgical Assessment of Epilepsy. clinicaltrials.gov; 2025. Accessed August 16, 2025. https://clinicaltrials.gov/study/NCT03893916
- Shammas A. Combined Role of PET and MEG in Nonlesional Epilepsy in Pediatric Population. clinicaltrials.gov; 2014. Accessed August 16, 2025. https://clinicaltrials.gov/study/NCT00741559

- **Different outcome (n= 11)**

- Wang Y, Liu M, Zheng W, et al. Causal Brain Network Predicts Surgical Outcomes in Patients With Drug-Resistant Epilepsy: A Retrospective Comparative Study. IEEE Trans Neural Syst Rehabil Eng. 2024;32:2719-2726. doi:10.1109/TNSRE.2024.3433533
- Li Z, Zhao B, Hu W, et al. Practical measurements distinguishing physiological and pathological stereoelectroencephalography channels based on high-frequency oscillations in the human brain. Epilepsia Open. 2024;9(4):1287-1299. doi:10.1002/epi4.12950
- Costa F, Schaft EV, Huiskamp G, et al. Robust compression and detection of epileptiform patterns in ECoG using a real-time spiking neural network hardware framework. Nat Commun. 2024;15(1):3255. Published 2024 Apr 16. doi:10.1038/s41467-024-47495-y
- Sun L, Feng C, Zhang E, et al. High-performance prediction of epilepsy surgical outcomes based on the genetic neural networks and hybrid iEEG marker. Sci Rep. 2024;14(1):6198. Published 2024 Mar 14. doi:10.1038/s41598-024-56827-3
- Hernández-Nava G, Salazar-Colores S, Cabal-Yepez E, Ramos-Arreguín JM. Parallel Ictal-Net, a Parallel CNN Architecture with Efficient Channel Attention for Seizure Detection. Sensors (Basel). 2024;24(3):716. Published 2024 Jan 23. doi:10.3390/s24030716
- Miron G, Müller PM, Holtkamp M, Meisel C. Prediction of epilepsy surgery outcome using foramen ovale EEG - A machine learning approach. Epilepsy Res. 2023;191:107111. doi:10.1016/j.eplepsyres.2023.107111
- Zhang Y, Chung H, Ngo JP, et al. Characterizing physiological high-frequency oscillations using deep learning. J Neural Eng. 2022;19(6):10.1088/1741-2552/aca4fa. Published 2022 Dec 7. doi:10.1088/1741-2552/aca4fa
- Park DK, Kim W, Thornburg OS, et al. Convolutional neural network-aided tuber segmentation in tuberous sclerosis complex patients correlates with electroencephalogram. Epilepsia. 2022;63(6):1530-1541. doi:10.1111/epi.17227
- Yamamoto S, Yanagisawa T, Fukuma R, et al. Data-driven electrophysiological feature based on deep learning to detect epileptic seizures.

J Neural Eng. 2021;18(5):10.1088/1741-2552/ac23bf. Published 2021 Sep 30. doi:10.1088/1741-2552/ac23bf

- Centracchio J, Sarno A, Esposito D, et al. Efficient automated localization of ECoG electrodes in CT images via shape analysis. Int J Comput Assist Radiol Surg. 2021;16(4):543-554. doi:10.1007/s11548-021-02325-0
- Dümpelmann M, Jacobs J, Kerber K, Schulze-Bonhage A. Automatic 80-250Hz "ripple" high frequency oscillation detection in invasive subdural grid and strip recordings in epilepsy by a radial basis function neural network. Clin Neurophysiol. 2012;123(9):1721-1731. doi:10.1016/j.clinph.2012.02.072
